# Microwave Radiometry at Frequencies From 500 to 1400 MHz: An Emerging Technology for Earth Observations

**DOI:** 10.1109/jstars.2021.3073286

**Published:** 2021-04-14

**Authors:** Joel T. Johnson, Kenneth C. Jezek, Giovanni Macelloni, Marco Brogioni, Leung Tsang, Emmanuel P. Dinnat, Jeffrey P. Walker, Nan Ye, Sidharth Misra, Jeffrey R. Piepmeier, Rajat Bindlish, David M. LeVine, Peggy E. O’Neill, Lars Kaleschke, Mark J. Andrews, Caglar Yardim, Mustafa Aksoy, Michael Durand, Chi-Chih Chen, Oguz Demir, Alexandra Bringer, Julie Z. Miller, Shannon T. Brown, Ron Kwok, Tong Lee, Yann Kerr, Dara Entekhabi, Jinzheng Peng, Andreas Colliander, Steven Chan, Joseph A. MacGregor, Brooke Medley, Roger DeRoo, Mark Drinkwater

**Affiliations:** ElectroScience Laboratory, The Ohio State University, Columbus, OH 43212 USA; School of Earth Sciences, The Ohio State University, Columbus, OH 43210 USA; Department of Remote Sensing, Nello Carrara Institute of Applied Physics National Research Council, 50019 Sesto Fiorentino, Italy; Department of Remote Sensing, Nello Carrara Institute of Applied Physics National Research Council, 50019 Sesto Fiorentino, Italy; University of Michigan, Ann Arbor, MI 48109 USA.; Cryospheric Sciences Lab, NASA GSFC/Chapman University, Greenbelt, MD, 20771 , USA; Dept. of Civil Engineering, Monash University, Clayton, VIC, Australia; Dept. of Civil Engineering, Monash University, Clayton, VIC, Australia; NASA Jet Propulsion Laboratory, Pasadena, CA 91109 USA; NASA Goddard Space Flight Center, Greenbelt, MD 20771 USA; NASA Goddard Space Flight Center, Greenbelt, MD 20771 USA; NASA Goddard Space Flight Center, Greenbelt, MD 20771 USA; NASA Goddard Space Flight Center, Greenbelt, MD 20771 USA; Department of Sea Ice Physics, Alfred-Wegener-Institut für Polar und Meeresforschung, 27570 Bremerhaven, Germany; ElectroScience Laboratory, The Ohio State University, Columbus, OH 43212 USA; Electrical and Computer Engineering Department, Ohio State University, Columbus, OH 43212 USA; Electrical and Computer Engineering Department, University at Albany, Albany, NY 12222 USA; School of Earth Sciences, The Ohio State University, Columbus, OH 43210 USA; ElectroScience Laboratory, The Ohio State University, Columbus, OH 43212 USA; ElectroScience Laboratory, The Ohio State University, Columbus, OH 43212 USA; Electrical and Computer Engineering Department, Ohio State University, Columbus, OH 43212 USA; ElectroScience Laboratory, The Ohio State University, Columbus, OH 43212 USA; Electrical and Computer Engineering Department, Ohio State University, Columbus, OH 43212 USA; Earth Observations, Cooperative Institute for Research in Environmental Sciences, Boulder, CO 80309 USA; NASA Jet Propulsion Laboratory, Pasadena, CA 91109 USA; Polar Science Center, University of Washington Applied Physics Laboratory, Seattle, WA 98105 USA; NASA Jet Propulsion Laboratory, Pasadena, CA 91109 USA; CNES, CESBIO, Toulouse Cedex 9, France; MIT, Cambridge, MA 02139 USA; NASA Goddard Space Flight Center, Greenbelt, MD 20771 USA; NASA Jet Propulsion Laboratory, Pasadena, CA 91109 USA; NASA Jet Propulsion Laboratory, Pasadena, CA 91109 USA; NASA Goddard Space Flight Center, Greenbelt, MD 20771 USA; NASA Goddard Space Flight Center, Greenbelt, MD 20771 USA; University of Michigan, Ann Arbor, MI 48109 USA.; Mission Science Division, ESA-ESTEC, 2201 AZ Noordwijk, the Netherlands

**Keywords:** Earth observations, microwave radiometry

## Abstract

Microwave radiometry has provided valuable spaceborne observations of Earth’s geophysical properties for decades. The recent SMOS, Aquarius, and SMAP satellites have demonstrated the value of measurements at 1400 MHz for observing surface soil moisture, sea surface salinity, sea ice thickness, soil freeze/thaw state, and other geophysical variables. However, the information obtained is limited by penetration through the subsurface at 1400 MHz and by a reduced sensitivity to surface salinity in cold or wind-roughened waters. Recent airborne experiments have shown the potential of brightness temperature measurements from 500–1400 MHz to address these limitations by enabling sensing of soil moisture and sea ice thickness to greater depths, sensing of temperature deep within ice sheets, improved sensing of sea salinity in cold waters, and enhanced sensitivity to soil moisture under vegetation canopies. However, the absence of significant spectrum reserved for passive microwave measurements in the 500–1400 MHz band requires both an opportunistic sensing strategy and systems for reducing the impact of radio-frequency interference. Here, we summarize the potential advantages and applications of 500–1400 MHz microwave radiometry for Earth observation and review recent experiments and demonstrations of these concepts. We also describe the remaining questions and challenges to be addressed in advancing to future spaceborne operation of this technology along with recommendations for future research activities.

## I. Introduction

Microwave radiometry provides valuable observations of Earth’s geophysical properties, including those of the atmosphere, ocean, cryosphere, and land [1], [2]. Microwave radiometers observe the thermal noise naturally emitted from the observed scene, with the thermal noise power received reported in terms of the brightness temperature. This brightness temperature depends not only on the physical temperature of the medium observed (to which sensitivity is achieved approximately to the depth of microwave penetration), but also on the dielectric properties and surface roughness of the scene. Dielectric properties typically dominate the observed response, making microwave radiometry useful for sensing geophysical properties that affect the microwave permittivity, especially the presence of water. Further improvements in sensing performance can be achieved by combining measurements in multiple frequencies, incidence angles, or polarizations. Numerous spaceborne microwave radiometers have provided valuable geophysical observations on Earth’s surface through the use of brightness temperature measurements at frequencies near 1.4, 6.8, 10.7, 18.7, 22, 37, and 89 GHz, with additional sensors focused on atmospheric observations at even higher frequencies.

Because microwave radiometry involves measurement of the naturally emitted thermal noise power (which occurs at very small power levels), it is often performed in portions (or bands) of the electromagnetic spectrum where anthropogenic radio transmissions are restricted [2]. Despite these restrictions, no band is completely free of emissions from active services, due to the presence of both in-band (shared) and out-of-band signals. When such transmissions are present, they represent radio-frequency interference (RFI) to a microwave radiometer and can prevent or bias brightness temperatures measurements, potentially resulting in erroneous retrievals of geophysical products. Because RFI is often observed even in protected bands (e.g., [3], [4]), vigilant protection and enforcement of existing spectrum allocations are crucial. Many microwave-radiometer science applications benefit from the use of additional spectrum to improve radiometric performance, and measurements are often performed in portions of the spectrum that are not protected. This opportunistic use of the spectrum has been successful in some cases [2], [5] but is being compromised as radio-spectrum occupancy continues to increase.

To address the challenge of RFI, additional subsystems have been developed that aim to separate man-made signals from thermal emission contributions (e.g., [2]–[13]). These signal detection and RFI filtering approaches can in some cases allow brightness temperature observations to continue in nonprotected portions of the spectrum. However, an overall degradation occurs as compared to the case of fully available spectrum because corrupted portions of the observed time-frequency space must be discarded when estimating the brightness temperature of the observed scene.

The advancement of RFI filtering techniques has recently enabled the consideration of microwave radiometric measurements in the even more heavily occupied portions of the radio spectrum below the protected 1400–1427 MHz band [14]–[31]. While the high presence of radio transmissions in this portion of the spectrum represents a significant challenge, multiple experiments [14], [15],[22], [23],[26],[28], [29] over the past decade have demonstrated the value of passive microwave remote sensing at frequencies lower than 1400 MHz. An expansion into this lower frequency range opens the door to “hyperspectral” radiometry in which the brightness temperature is measured as a function of frequency instead of in a single narrowband channel alone. This further opens the possibility of new retrieval approaches in which information on geophysical properties is derived from a scene’s microwave spectral signature, analogous to retrievals performed in hyperspectral optical or infrared sensing.

This article reviews the motivations for the use of microwave radiometry at frequencies from 500 tο 1400 MHz (Section II), along with the associated technical challenges (Section III) including the significant presence of RFI and the requirement of large antenna sizes for spaceborne operation. Recent progress in demonstrating these approaches is then reviewed in Section IV, along with recommendations to achieve continued progress (Section V).

## II. Properties of Microwave Thermal Emission at 500–1400 MHz

A key factor that motivates microwave radiometry in this frequency range is the increased penetration through geophysical media (e.g., soil, vegetation, and ice) that occurs as the electromagnetic frequency is reduced and the wavelength increases. Lower frequencies also reduce sensitivity to surface roughness and to scattering from inhomogeneities within the medium observed. The measurement of the brightness temperature as a function of frequency is also of interest for a variety of remote sensing applications, as described below for specific terrestrial surfaces. The discussions to follow are derived from relatively low-order models that are sufficient to illustrate the basic physical properties expected for the 500–1400 MHz brightness temperatures of the media considered.

### A. Soil

**Fig. 1.**
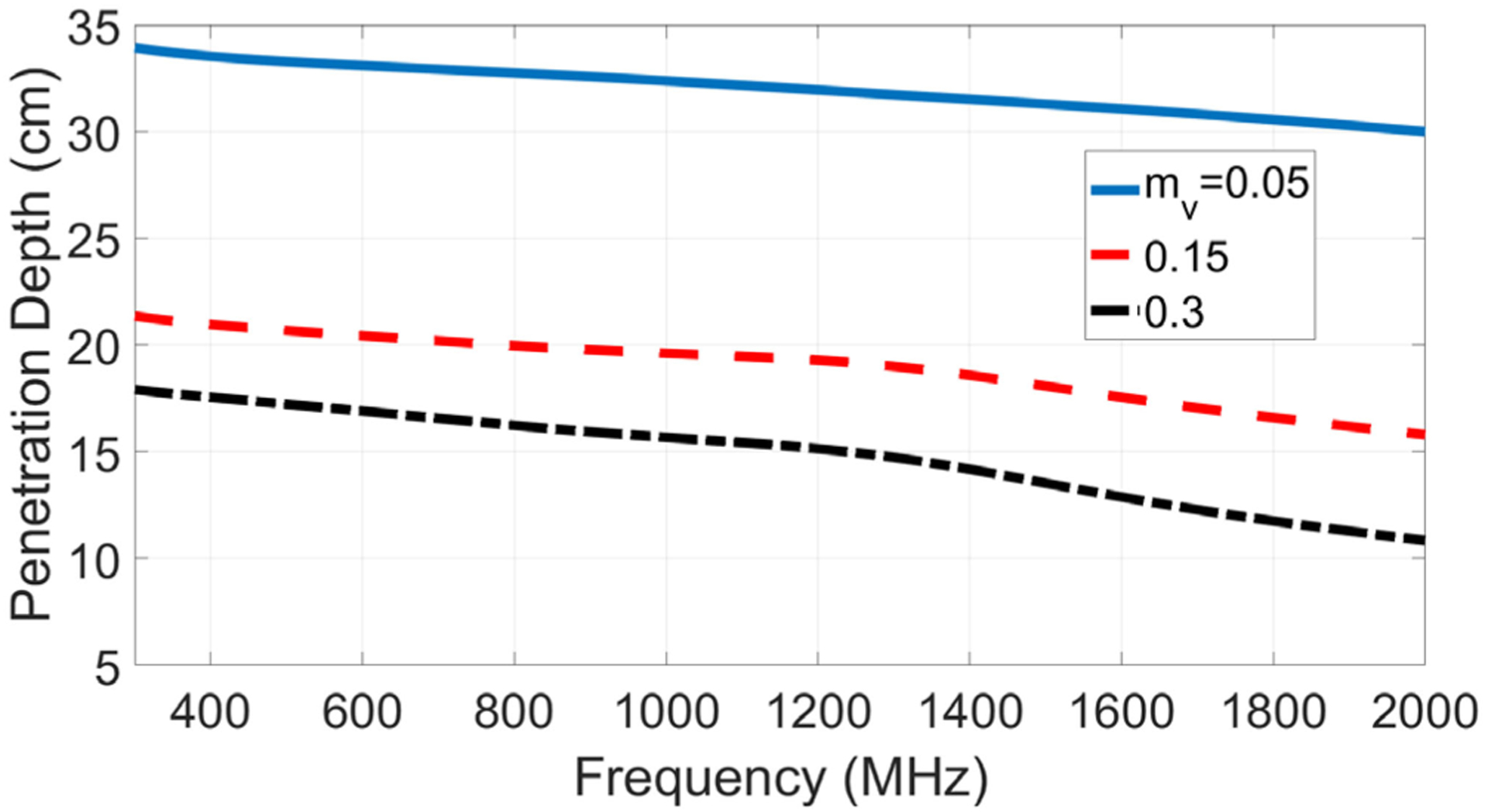
Penetration depths in a soil medium (20% sand, 60% clay, and bulk density 1.57 g/cm^3^) as a function of frequency and volumetric soil moisture (*m*_*v*_) using the dielectric model of [32].

Fig. 1 illustrates the penetration depth (i.e., the distance over which thermally emitted fields within the medium will propagate before attenuating by a factor of 1/*e*) as a function of frequency for a soil medium consisting of 60% clay and 20% sand of bulk density 1.57 g/cm^3^, obtained from the Peplinski model of the soil dielectric constant [32]. Curves are illustrated for three levels of soil moisture ranging from “dry” (volumetric soil moisture of 0.05 cm^3^/cm^3^) to “wet” (0.30 cm^3^/cm^3^). The increased penetration depths available at lower frequencies are apparent. Current 1400–1427 MHz soil moisture sensing using the SMAP or SMOS missions [33], [34] is described as being sensitive to soil moisture in the upper 5 cm of the surface when observed at an incidence angle of 40° (note Fig. 1 corresponds to sensing at nadir). The greater penetration available at lower frequencies enables sensing of soil moisture at greater depths [14], [15], and the possibility of sensing a soil moisture profile is also evident if measurements at multiple frequencies are combined (since individual frequencies will respond to the soil moisture only up to their approximate depth of penetration).

**Fig. 2.**
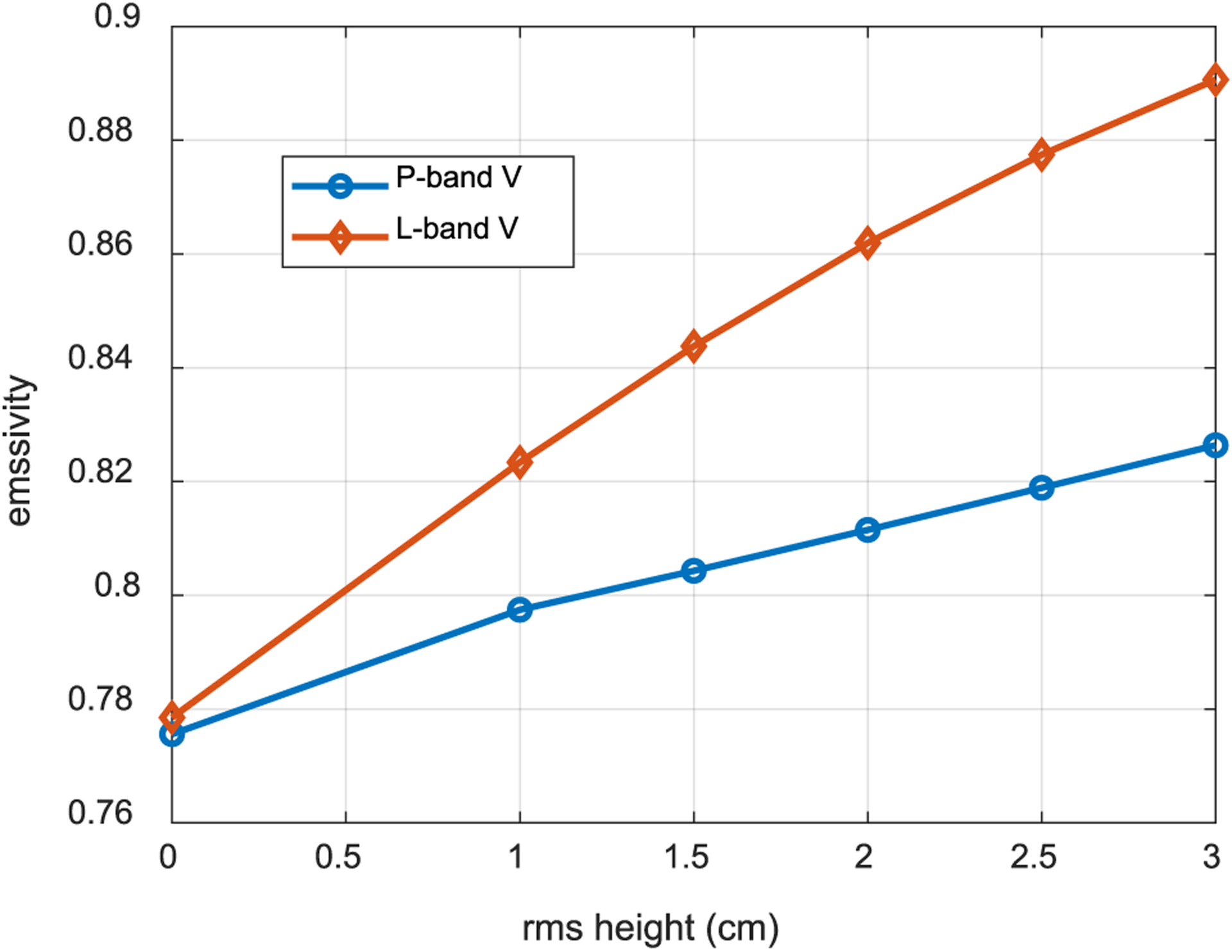
Vertically polarized (*V*) soil surface emissivity at 30° from zenith as a function of surface rms height (correlation length is 10 times the rms height) at 500 (P-band) and 1400 MHz (L-band).

The increased penetration depths that occur in this frequency range have also motivated the use of 225–500 MHz radar [35], [36] and specular reflections [37]–[39] for monitoring soil moisture at greater depths. All of these emerging technologies have the potential to extend the depths to which soil moisture can be sensed, with each sensing type (microwave radiometry as discussed here, radar backscatter, and specular reflection sensing) having distinct dependencies on other confounding parameters such as surface roughness and vegetation coverage. Fig. 2 examines the effect of surface roughness on microwave radiometry by plotting the vertically polarized emissivity of a simulated soil surface as a function of the surface root-mean-square height. These predictions were produced using fully 3-D numerical simulations of rough surface emissivity with the sparse-matrix canonical grid method [40]–[42]. The results compared for 500 and 1400 MHz illustrate the weaker impact of surface roughness at lower frequencies, so that this confounding factor is less likely to introduce errors that require subsequent correction in soil moisture retrievals.

Although the effects of vegetation on emissions at frequencies lower than 1400 MHz have been less studied, attenuation through a vegetation canopy should still be expressible at these frequencies in terms of an optical depth *τ* that satisfies *τ ≈ b VWC* [43], where VWC represents the vegetation water content in kg/m^2^. Previous studies of variations in vegetation optical depth (VOD) with frequency have suggested that the scaling coefficient *b* has the form *c/λ*^*x*^ with *c* and *x* constant and *λ* the electromagnetic wavelength. Van De Griend and Wigneron [44] report *x* values that vary from 0.4 to 1.4 depending on the vegetation type, so that VOD reductions at 500 MHz as compared to 1400 MHz can be estimated to range from 30%–75%.

**Fig. 3.**
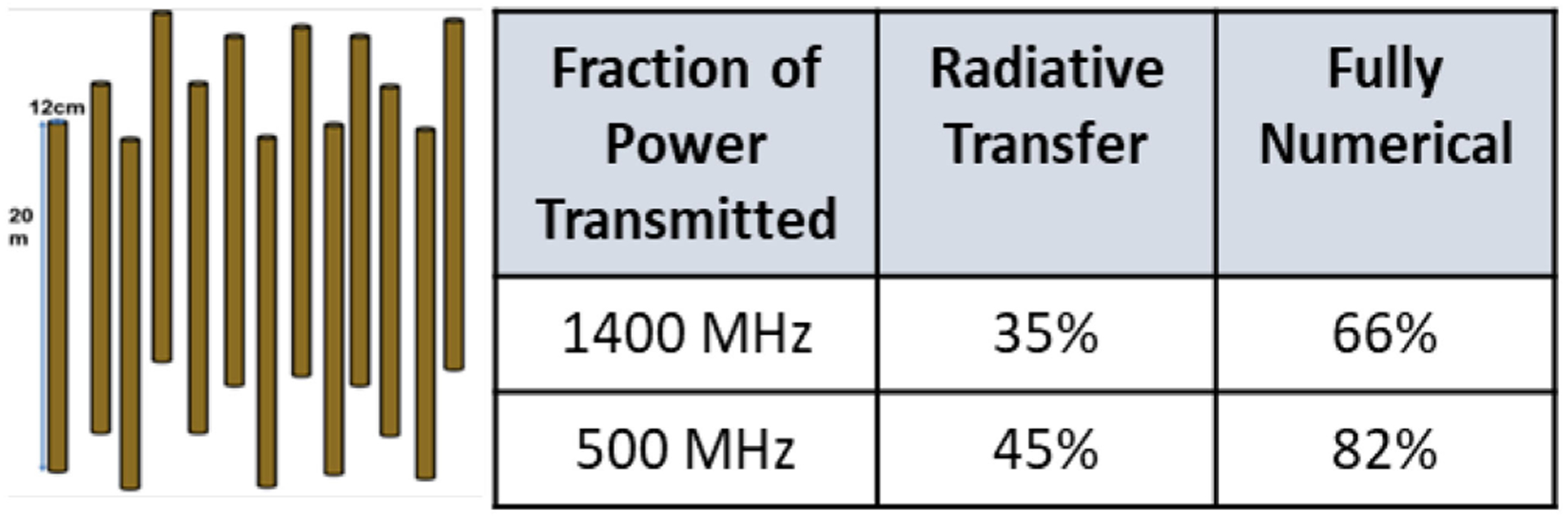
Simulated vegetation medium comprised of 196 dielectric cylinders (left) for which transmission through the medium (right) was computed at 500 and 1400 MHz using both a traditional radiative transfer approach and a fully numerical approach.

Further evidence of the improved penetration through vegetation at lower frequencies was obtained through a numerical simulation of transmission through a simulated vegetation medium representing forest trees [45], [46]. The simulated medium consists of 196 cylinders of 20 m height and 12 cm diameter arranged as shown in Fig. 3. Cylinder permittivities were determined using a model related to the VWC, with a resulting VWC of 17.3 kg/m^2^ for the simulated medium. The fraction of the power density from an illuminating plane wave transmitted through the medium was computed at both 500 and 1400 MHz using a fully numerical solution as well as a traditional radiative transfer/distorted Born approximation method. The results, though for a specific geometry only, show the increased penetration that occurs at 500 MHz that is predicted by the numerical model to be even larger due to its more accurate consideration of the medium geometry for this specific case (Fig. 3).

The accuracy that has been demonstrated for soil moisture sensing using the 1400 MHz microwave radiometry of the SMAP and SMOS missions motivates the examination of the potential of microwave radiometry at lower frequencies for soil moisture monitoring; demonstrations of this concept will be described in Section IV. The increased penetration depths available also suggest the application of these methods to the sensing of permafrost properties [47], in which monitoring the status of the seasonally thawed “active layer” above deeper frozen soils is of interest. Both the SMOS and SMAP L-band radiometers have demonstrated the capability of sensing frozen soil to a depth of 15 cm for monitoring freeze/thaw state [48], [49]. Lower frequencies penetrate further into permafrost, and the use of multiple-frequency measurements suggests the capability of observing permafrost subsurface properties as a function of depth to monitor the status of the active layer.

### B. Sea Surfaces

Although penetration into seawater remains small even at frequencies as low as 500 MHz, the increased impact of sea water conductivity at lower frequencies results in an increased sensitivity to sea surface salinity (SSS, [16]–[18]). This is particularly valuable for colder waters, where past 1400 MHz microwave radiometers such as Aquarius, SMOS, and SMAP have experienced challenges with achieving accurate estimation of SSS [18].

**Fig. 4.**
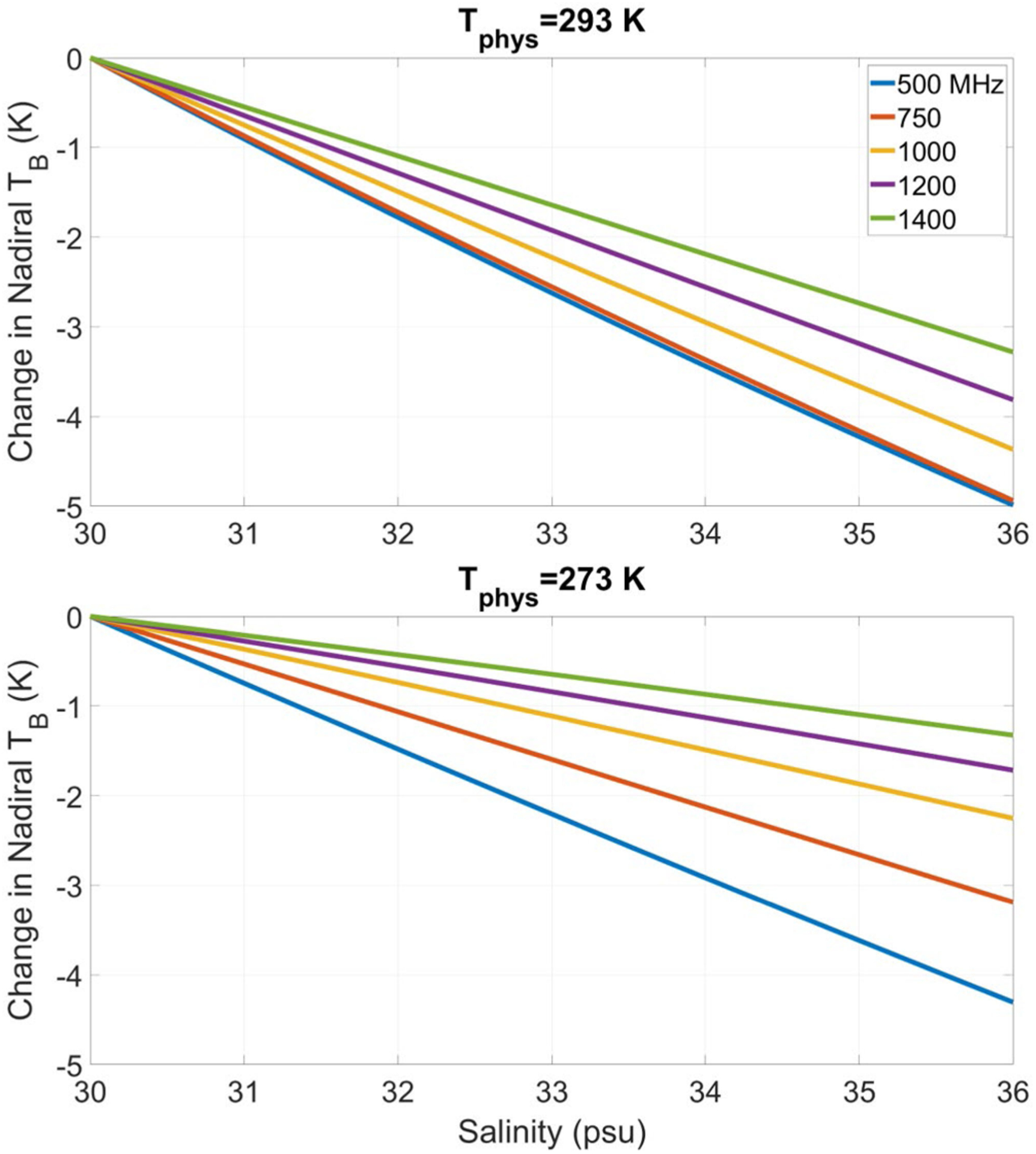
Change in nadir brightness temperature (*T*_*B*_) from 30 psu for a flat sea surface as a function of salinity and frequency for sea water temperatures of 20°C (upper) and 0°C (lower).

Fig. 4 illustrates these behaviors using the sea water dielectric constant model of [50] (see also [51], [52] that show similar variations with salinity and temperature). Changes in the predicted nadir brightness temperatures for a flat sea surface are shown as a function of frequency and salinity at sea surface temperatures (SST) of 20°C and 40°C. The changes are plotted with respect to the predicted brightness temperature at SSS 30 psu to highlight the sensitivity to changes in SSS. The results demonstrate the increased sensitivity achieved at lower frequencies under both “warm” and “cold” conditions. Note that the 0°C 500 MHz change in brightness temperature with SSS remains larger than that at 1400 MHz at 20°C. The challenge in sensing SSS remotely in cold waters using 1400 MHz alone is also evident, given that a change of only ~1.2 K in nadir brightness temperatures occurs as SSS varies from 30–36 psu. At 500 MHz, this change is three times larger (i.e., 4.5 K).

As in the soil surface case, lower microwave frequencies will experience a decreased sensitivity to surface roughness, reducing their utility for sensing oceanic wind speeds while increasing utility for sensing SSS. The signatures of salinity, wind speed, sea-surface temperature, and galactic emissions also vary over the 500–1400 MHz range, potentially reducing the requirement for ancillary data in SSS retrievals if brightness temperatures are measured as a function of frequency.

### C. Sea Ice

The strong contrast in microwave thermal emission between open water and ice covered ocean surfaces has long enabled the monitoring of sea ice coverage using microwave radiometry [53]. Because the presence of ice can be detected using a wide range of microwave frequencies, the primary utility of frequencies at or lower than 1400 MHz lies in the sensing of other ice properties of interest in climate studies, such as ice thickness [19]–[23].

The success of the SMOS and SMAP 1400-MHz radiometers in providing sea ice thickness information at thicknesses up to ~50 cm [54]–[56] motivates examination of the benefits of lower frequencies. Similar expectations regarding increased penetration occur for sea ice media, although in this case the complexity of the sea ice medium must be considered [20]–[23], [57]. In particular, the wide variation in salinity that can occur between first year and multiyear ice types and their overlying snow cover significantly influences penetration into sea ice at all frequencies.

**Fig. 5.**
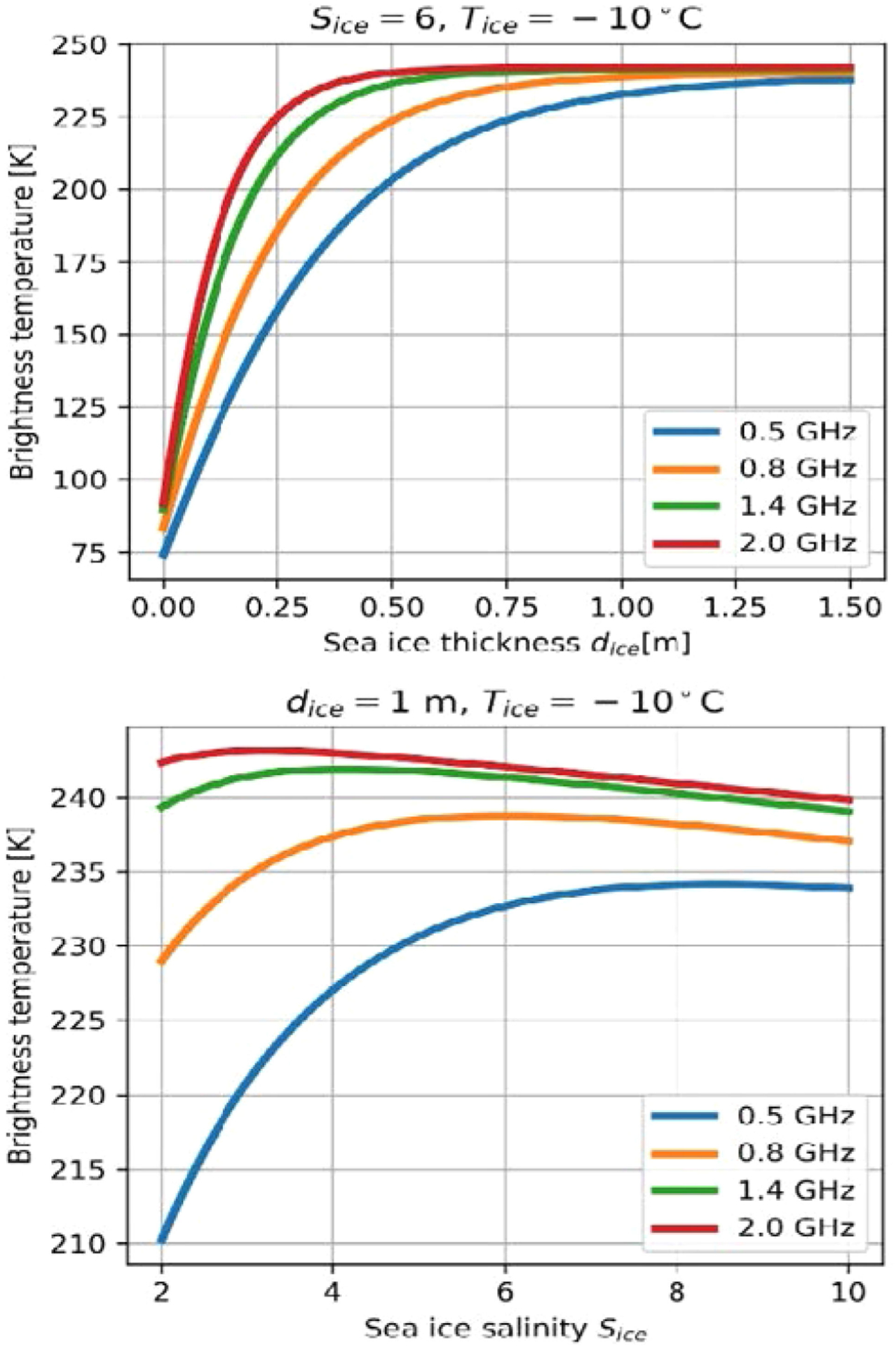
Modeled sea ice nadiral brightness temperatures versus ice thickness (upper) and salinity in psu (lower) and frequency. Ice physical temperature of −10 C, salinity 6 psu (upper), and thickness 1 m (lower).

Fig. 5 illustrates the relevant effects using models of sea ice thermal emission [22], [57]. The upper plot illustrates predicted nadir brightness temperatures as a function of ice thickness (for ice salinity 6 psu and physical temperature −10°C) and frequency, while the lower plot considers brightness temperatures versus salinity for ice temperature −10°C and a fixed 1-m ice thickness. Results were computed using a radiative transfer approach [22]; the predictions of 3-D fully numerical solution ([40]–[42], not shown) that includes surface roughness effects have also been investigated. The results in Fig. 5 demonstrate the saturation in 1400-MHz brightness temperatures that occurs for ice thicknesses greater than ~50 cm, so that sensitivity to thickness is reduced beyond this range. In contrast, brightness temperatures at 500 MHz retain sensitivity to ice thickness even for thicknesses greater than 1 m. The variations in brightness temperatures with salinity also indicate the potential for ambiguities in the retrieval process if a single-frequency measurement is performed (because the same brightness temperature can occur for two distinct salinity values); however, the use of multiple-frequency channels can resolve such ambiguities and can enable the sensing of salinity.

Improving the remote sensing of sea ice thickness in the 0.5–1.5-m range is of particular interest, given the challenges faced for this thickness range [55] by current and planned sensors (i.e., 1400-MHz radiometers, laser, and radar altimeters). The increasing presence of seasonal sea ice in this thickness range—due to the continuing decline of perennial ice in the Arctic [58]—further motivates a focus on developing the ability to remotely measure and monitor the thickness of younger sea ice and on bridging the gap in thickness sensing between the “thinner ice” performance available from 1400 MHz radiometry and the “thicker ice” performance available from radar or optical altimetry.

Lower frequencies are also more sensitive to ice salinity, suggesting the possibility that measurements at multiple frequencies could be used to sense both ice thickness and salinity simultaneously [22], [59]. Studies of the effects of the roughness of the sea ice/water interface using a 3-D fully numerical solution also support the hypothesis that surface roughness effects are reduced at lower frequencies. In addition, the potential for sensing the thickness of both sea ice and any covering snow layer (neglected in Fig. 5) has also been described [60], [61]. Additional discussions of this possibility are provided in Section IV.

### D. Ice Sheets

**Fig. 6.**
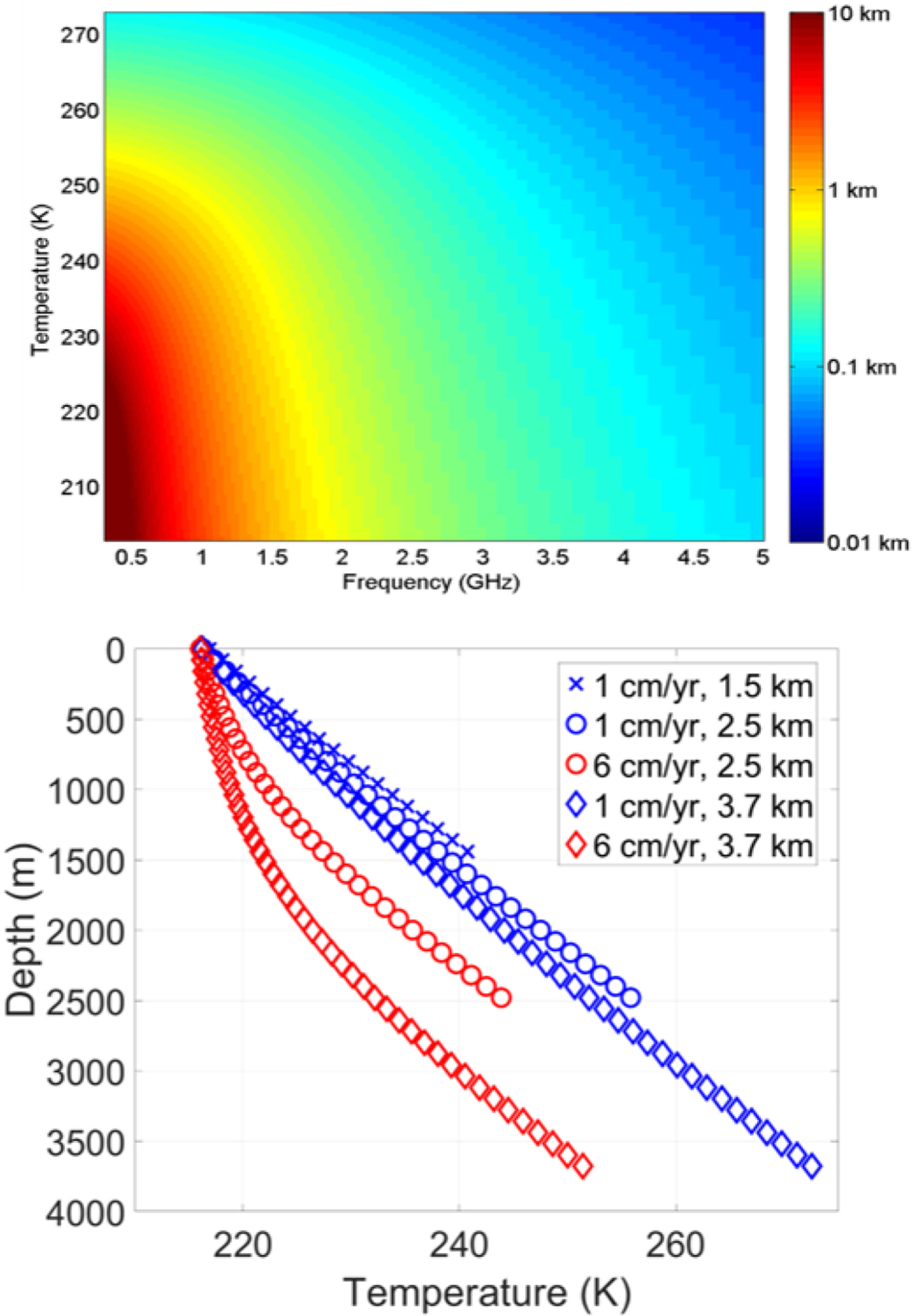
(Upper) Penetration depth in laboratory-grown pure ice as a function of frequency and ice temperature (lower) example temperature profiles within an ice sheet under the Robin model [67], [68] for varying surface accumulation rates and ice thicknesses and surface temperature 216 K.

Fig. 6 provides an illustration of penetration depths in pure ice as a function of frequency and ice temperature following the model of [62]. This model indicates that penetration depths of multiple kilometers can occur in cold and dry (polar) ice at frequencies less than 1000 MHz, an inference that is well supported by decades of radar sounding of ice sheets at frequencies from 1 to 1000 MHz [63]. Because the thermal emission observed by a microwave radiometer should be sensitive to emissions from portions of the ice sheet within the penetration depth, the potential for remotely sensing the temperatures within an ice sheet arises [24]–[28], [64]–[66]. Macelloni *et al.* [66] explore this possibility using the 1400 MHz observations of SMOS, and demonstrates the limitations associated with the use of a single-frequency band for this application. Improvements are expected if measurements are performed at multiple frequencies, because the depth of penetration varies with frequency.

The lower portion of Fig. 6 illustrates this concept further. Multiple example ice-sheet internal temperature profiles are shown, generated using a simple 1-D model [67], [68]. The increasing temperature with depth arises because the ice sheet effectively insulates the bedrock below from the cold surface. The resulting temperature profile is a balance between down-ward vertical advection of cold ice from the surface and slow conductive warming from the geothermal heat flux at the ice-sheet base. Ice-sheet brightness temperatures should therefore be larger at lower frequencies because these frequencies are sensitive to the typically higher physical temperatures deeper within the ice sheet. Measurements at higher frequencies should correspond to the lower physical temperatures at shallower depths. By combining multiple-frequency measurements into a model-based retrieval, information on the temperature profile should be achievable [24]–[28]. More information on this approach is provided in Section IV.

The above discussion neglected inhomogeneities within the ice sheet. While the 500–1400 MHz band is not expected to be sensitive to the grain size of snow particles in the upper firn (a key parameter for microwave radiometry at higher frequencies such as 19 and 37 GHz), fluctuations in ice density with depth in the upper ~100 m do have a significant impact on thermal emission [25]–[28], [69]–[71]. Methods for addressing this confounding factor in sensing deep ice temperature are further described in Section IV. In addition to sensing the temperature profile, in some cases lower frequency brightness temperature measurements (e.g., at 500 MHz) may be sensitive to dielectric properties at the ice sheet base [24], potentially enabling detection of the presence of basal liquid water and thereby further improving the monitoring of conditions that affect ice sheet dynamics.

### E. Lake Ice and Snow Thickness Sensing

For media such as lake ice or snow covered land that can be modeled locally as a layered dielectric medium having planar interfaces on which all roughness is small compared to the electromagnetic wavelength, brightness temperatures can exhibit an oscillatory behavior in the spectrum [1], [72]–[78]. The oscillations in frequency arise from self-interference effects because reflections within the layered medium cause a portion of the emitted signals to transit through layers more than once. For sufficiently flat interfaces, these multipath emissions can interfere with the directly emitted signals. In the simplest geometry of a single dielectric layer, the spectrum exhibits alternating maxima and minima caused by constructive and destructive self-interference. The spectral spacing of these features is determined by the round-trip electrical length of the layer, and thereby provides a method, independent of the brightness temperature, to measure the layer thickness.

While this effect can be a confounding signal, it can also be an observable. This effect has not yet been observed below 1400 MHz, but it has been observed at 7–11 GHz in freshwater ice and in the snow on that ice [75]–[77] and is under investigation for snow sensing on land surfaces [78]. The effect has also been observed in soil surfaces at 1400 MHz [72] and from buried planar objects at 2–6 GHz [73]. The reduced volume and surface scattering at the longer wavelengths discussed here imply the presence of this signal below 1400 MHz and the potential application of this approach in sensing snow or ice layer thickness.

## III. Technical Challenges

While the previous section clearly motivates extending microwave radiometry into the 500–1400 MHz range, several challenges also exist that must be addressed to enable successful measurements.

### A. Aperture Sizes

A first concern is the increased aperture size required to retain a scientifically relevant spatial resolution when operating from a satellite. Because the spatial resolution of a microwave radiometer is determined by the size of its antenna relative to the wavelength, an increase in aperture dimension proportional to the wavelength used should be expected (i.e., a factor of 2.8 when going to 500 from 1400 MHz). This is true regardless of whether an interferometric or real aperture radiometry approach is used, although an interferometric system could permit use of a thinned array antenna that is capable of observing at multiple incidence angles simultaneously.

The 6-m antenna diameter of SMAP’s 1400-MHz radiometer [34] serves as a benchmark for further examination. This aperture size enables a 40-km Earth footprint when operating from SMAP’s orbit altitude of 685 km and observing at an Earth incidence angle of 40°. A similar system operating at 500 MHz would require an aperture size 280% larger, i.e., a diameter of 16.8 m. While deployable reflectors of this size have been reported [79], methods for reducing the required aperture size are highly desirable to simplify spacecraft accommodation and operation.

Options under consideration include operation at a reduced orbit altitude, e.g., a reduction to 400-km altitude would bring the required aperture diameter closer to 10 m. Performing measurements at nadir also would eliminate the extension of footprint diameter caused by the projection of the antenna beam pattern onto the Earth’s surface (proportional to $\frac{1}{\text{cos }\theta }$, where *θ* is the incidence angle). Operation at nadir could then reduce the required aperture dimension by a factor of approximately $\sqrt{\text{cos }40{}^{\circ}}$ (12.5% reduction) as compared to SMAP; the square root of the projection factor is considered since the projection applies for only one dimension of the footprint. Note, however, that operation near nadir reduces the utility of dual-polarization measurements and would likely reduce the observed swath width and spatial coverage because conical scanning would no longer be possible. These factors may be unacceptable for some applications, for example those requiring frequent revisit coverage near the equator.

A reduction in the desired spatial resolution from 40 km to a coarser resolution may also not be detrimental for some science investigations. In this regard, NASA’s Aquarius mission serves as an example, as its 1400-MHz microwave radiometer used an antenna diameter of 2.5 m for measurements of SSS at ~100 km spatial resolution [80].

Beyond considerations of size, the antenna must have the required effective aperture, beam width, and beam efficiency to be capable of meeting desired spatial resolution requirements over the range of frequencies of interest. Trade-offs of antenna gain and impedance matching as a function of frequency can then occur. New antenna concepts are available to address these challenges, including the use of end-fire antenna types deployed using extendable booms [81]–[83], the creation of an antenna array through the use of multiple spacecraft flying in formation [84], or new lightweight deployable aperture antenna types [79]. These developments make clear that current and emerging technologies are capable of overcoming the challenges of increased aperture size and wideband performance.

### B. RFI

The radio spectrum from 500 tο 1400 MHz is heavily occupied and used worldwide for television broadcast (~500–700 MHz), fixed and mobile communications (~700–960, 1350–1400 MHz), radio navigation (~960–1350 MHz), and a host of other applications [2]. These sources can cause RFI to a radiometer and can corrupt measurement of the underlying naturally emitted thermal signals.

While some approaches have been proposed for “estimating and subtracting” the man-made components of measured signals [85] so that the brightness temperature of the remaining signal can be obtained, the vast majority of brightness temperature sensing methods are based on the exclusion from use of any portions of the observed time, frequency, or angle space that contain RFI. The use of resolution in angle space implies measurements at multiple angles as acquired with a phased-array antenna [86]–[88], while resolution in time implies that multiple samples of observed signals are obtained within an integration period, and resolution in frequency implies that the radiometer bandwidth is divided into multiple subchannels [3]–[9]. The general approach is to estimate the total thermal noise power within a given integration time and observed bandwidth by integrating the power in all subtimes and subfrequency channels not flagged as containing RFI. A variety of detection algorithms have been described for the detection of RFI, including those based on signal time, frequency, angle, polarization, or statistical properties [3]–[7]. Because specific algorithms typically are designed for specific interferer types, combining the flags of multiple detection approaches is required to improve performance; this approach is implemented in the RFI processing for SMAP’s radiometer [3], [4]. Recent demonstrations have also shown the ability to perform RFI detection and filtering in real-time on-board a spacecraft, so that an increased data rate is not required for downlinking all time and frequency subsamples for use in ground processing [10]–[13]. These detection and filtering approaches work well when interference is sparse in the observation space. A recent experiment, however, demonstrated that such methods can fail in persistently shared spectrum [89].

Regardless of the detection approaches used, the loss of time and frequency samples that results following RFI flagging causes a degradation in the radiometric resolution, commonly described in terms of the noise equivalent delta temperature (NEDT):

(1) $$\text{NEDT }\propto \frac{T_{\text{sys}}+T_{\text{scene}}}{\sqrt{N}}$$

where *T*_sys_ and *T*_scene_ represent the receiver temperature and scene brightness temperatures, respectively, and *N* ≈ (BW)*t*_int_ is the effective number of averaged independent samples, where BW is the radiometer bandwidth and *t*_int_ the integration time. Because RFI flagging reduces *N* by removing time and frequency samples from the integration process, NEDT is increased. However, the degradation is relatively slow: for example, flagging 75% of the time and frequency samples within an integration period degrades NEDT by a factor of only two. These considerations suggest that microwave radiometers operating at 500–1400 MHz should be designed to achieve NEDT values that are a factor of two or more better than the NEDT corresponding to the desired science goals if no RFI is present, so that degradations in this parameter caused by RFI can still be tolerated. Although the radiometric uncertainty values required to achieve a specific performance for a particular geophysical product remain under investigation, preliminary results for ice sheet temperature profile sensing [25], [26] and for sea ice thickness sensing [59] have shown that radiometric uncertainties in the range 0.5–1 K when achieved in multiple-frequency channels in the 500–1400 MHz range can provide desirable science performance.

The success of microwave radiometry in unprotected bands depends largely on the assumption that the available time–frequency space within such bands is not fully utilized, so that microwave emission measurements of value to scientific applications still be made within temporarily unused portions. How valid is this assumption? Some information can be obtained by considering the properties of systems already operating in this portion of the spectrum. For example, broadcast television already operates on the principle of sharing in frequency through the licensing process. In a given location, broadcasters are required to have sufficiently separated frequencies to prevent interference, so that unused portions of the spectrum occur. This occupancy then varies from location to location, but on average a significant portion of the spectrum can remain available. Similar considerations arise for radar-based radio-navigation systems, whose pulsed transmission types produce significant RFI only for a portion of an integration time that is readily detected and filtered. The reduced presence of transmitters in locations with smaller human populations (e.g., high latitudes or other remote locations where remote sensing has greatest benefit) also suggests that successful thermal emission measurements should be possible in these regions. The particular benefits of lower frequency measurements in high latitude regions (for example, to observe sea ice, ice sheets, permafrost, or high-latitude sea salinity) were discussed in the previous section. Examples illustrating successful measurement of 500–1400 MHz thermal emission will be shown in Section IV.

These arguments in no way impact the importance of retaining protected portions of the spectrum for microwave radiometric measurements, as it is only in such bands that scientific performance can be guaranteed globally, and it is only through the use of such bands that the accuracy and precision of the proposed 500–1400 MHz radiometric measurements can be confirmed. We merely highlight the scientific opportunities that lie within other portions of the spectrum when permitted by the instantaneous spectral occupancy of the region under observation.

### C. Receiver Design and Calibration

Many of the sensing applications described in Section II benefit from the measurement of thermal emissions as a function of frequency. The requirement to exploit differing portions of the spectrum opportunistically also motivates operation over a range of frequencies. “Wide-band” operation implies that the radiometer receiver should provide multiple-frequency channels observing over the 500–1400 MHz frequency range, in contrast to more traditional designs that use single-frequency receivers (or multiple single-frequency receivers used across widely separated bands). Below we assume that the radiometer receiver includes an analog front-end subsystem for amplifying, filtering, down-converting, or conditioning the received RF signals appropriately, followed by a digital receiver that samples signals provided by the analog front-end for further processing and recording.

One option for achieving multiple-frequency measurements uses a “traditional” approach in which multiple single-frequency receivers are combined, each having its own front-end and subsequent receiver chain that receive signals from a common antenna. The simpler “narrow-band” design of each individual receiver simplifies component selection and improves RF performance, but comes at the cost of added complexity, mass, power consumption, and volume that grows with the number of channels. The digital receiver for such systems can consist either of a separate digital subsystem for each channel or a single digital receiver that samples the recombined outputs of multiple-frequency channels.

An alternative approach uses a wide-band receiver that has a single analog receiver for the entire bandwidth with measurements as a function of frequency computed by the digital receiver. The continuing growth in the performance of analog-to-digital (A/D) converters and digital processing subsystems has enabled the real-time processing of bandwidths 1 GHz or larger [10], [11], making this approach feasible with current technologies.

While both approaches are available (or their combination into a hybrid strategy), multiple considerations motivate the early separation of a received wider bandwidth into narrower bandwidth subchannels prior to digitization. A major concern arises from the potential corrupting effect of a very strong interferer on the analog portions of the receiver. Should an interferer’s power in a portion of the band be sufficient to cause a receiver component to saturate, any portion of the observed bandwidth encountering the saturated component will also be impacted. This suggests attempting to minimize the analog receiver gain in a wide bandwidth radiometer, as well as analog separation of channels prior to the highest gain stages of the receiver. Note that the separated channels could still be recombined in an analog fashion prior to their digitization.

Requirements for radiometer calibration [29], [30] also motivate separation into narrower frequency sub-bands in the analog domain. Microwave radiometer calibration methods are typically based on the assumption that impedance mismatch effects are small, so that the impact of internal reflections within the analog subsystem is minimal. In this case, component effects can be modeled by their impact on signal amplitudes only, with these effects typically remaining relatively stable over time and correctable using standard internal and external calibration methods.

When impedance mismatches become more significant, as is typical for wide bandwidth components, the phase interference effects that occur cause signals within the radiometer to depend on both the amplitude and phase of individual components and their interconnections. Because phase responses can vary more significantly with changes in temperature, vibration, or other effects, a stable radiometer calibration can be more difficult to achieve in the fully wideband radiometer case, unless analog components are minimized.

A variety of receiver architectures and calibration strategies have been demonstrated successfully, as will be described in Section IV, and the relative trade-offs of differing strategies remain a subject of active investigation [29], [30], [90], [91]. An alternative wide-band receiver implementation for measuring the thickness of layered media is also under investigation through the measurement of the time-domain autocorrelation of received thermal noise [74]–[78], although results from such systems in the 500–1400 MHz frequency range have yet to be reported. Finally, we note that current methods for “vicarious” external calibration based on expectations for the long-term behavior of the brightness temperature of Earth’s ocean or rain forest regions [92]–[95] will require the creation of new models for these effects at frequencies less than 1400 MHz.

### D. Ionospheric and Celestial Emission Effects

A final challenge for spaceborne operation arises from the increased influence of the ionosphere and celestial emission sources as frequency decreases.

From 500 tο 1400 MHz, ionospheric effects can still be represented using approximations valid at 1400 MHz, so that the primary factor governing ionospheric influence is the total electron content (TEC) along the path. In this frequency range, both the amount of Faraday rotation and ionosphere specific attenuation are inversely proportional to the frequency squared, so that both are ~8 times larger at 500 MHz as compared to 1400 MHz. The compensation of ionospheric contributions is therefore required to ensure accurate measurements. Continuing improvements in knowledge of the ionospheric TEC suggest that the required correction methods are available, as has been already demonstrated at 1400 MHz [96], [97]. The use of nadir observations in circular polarization, as proposed in [31], can also help to reduce the impact of ionospheric effects.

The impact of brightness contributions from celestial sources, as well as those from the sun and moon, must also be corrected to achieve accurate Earth brightness temperature estimates. Thermal emission contributions from many celestial sources increase at lower frequencies, making their contributions more significant than at 1400 MHz. The availability of sky maps [98], [99] for such sources and predictions of their radio emissions as a function of frequency makes the required corrections feasible with currently available information. It is noted that these effects are most significant for oceanic measurements, since the reflectivity of these emissions is most significant over the sea surface. The reduced impact of roughness at lower frequencies may also simplify the modeling required as compared to the reflected sky correction algorithms used at 1400 MHz [100], [101].

## IV. Recent Demonstrations

### A. Measurements of Ice Sheet Internal Temperatures

**Fig. 7.**
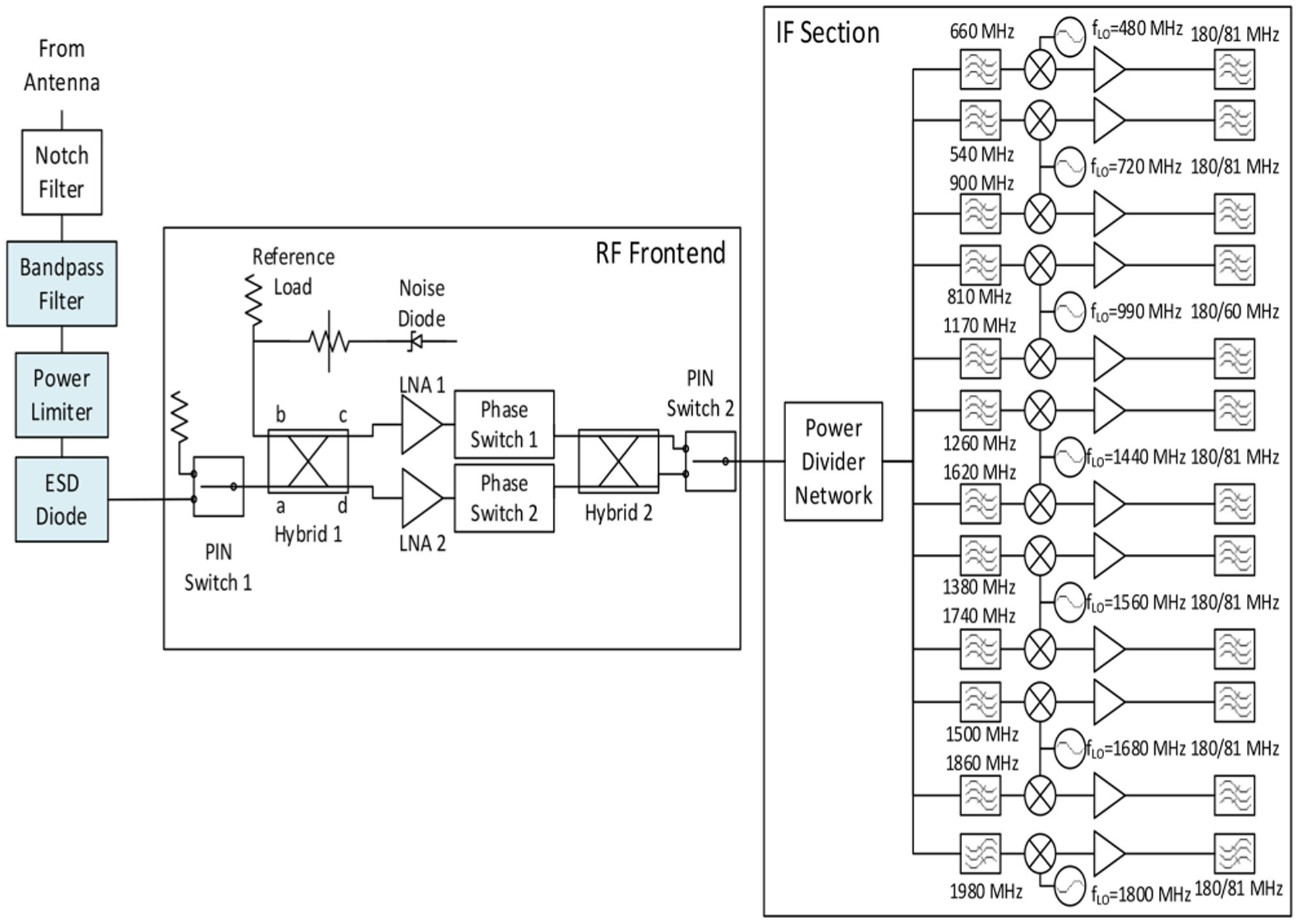
Schematic of the UWBRAD microwave radiometer.

Measurements of ice sheet and sea ice brightness temperatures from 500 to 2000 MHz are reported in [22], [26], [28], and [29] using the ultra-wideband software defined microwave radiometer (UWBRAD) of The Ohio State University. This instrument divides the observed spectrum into 12–100 MHz channels, with each channel further resolved into 512 subchannels as part of RFI detection and filtering operations. Table I provides a summary of the instrument properties. The radiometer design uses a “pseudo correlation”-type wideband front-end [91] that is then subdivided and filtered into subchannels before the down-conversion of each channel to IF center frequency 162 MHz and sampling at 250 MSPS (Fig. 7). Data sampling is performed by A/D converters interfaced to a computer, and all subsequent processing is performed by software in the computer.

**Fig. 8.**
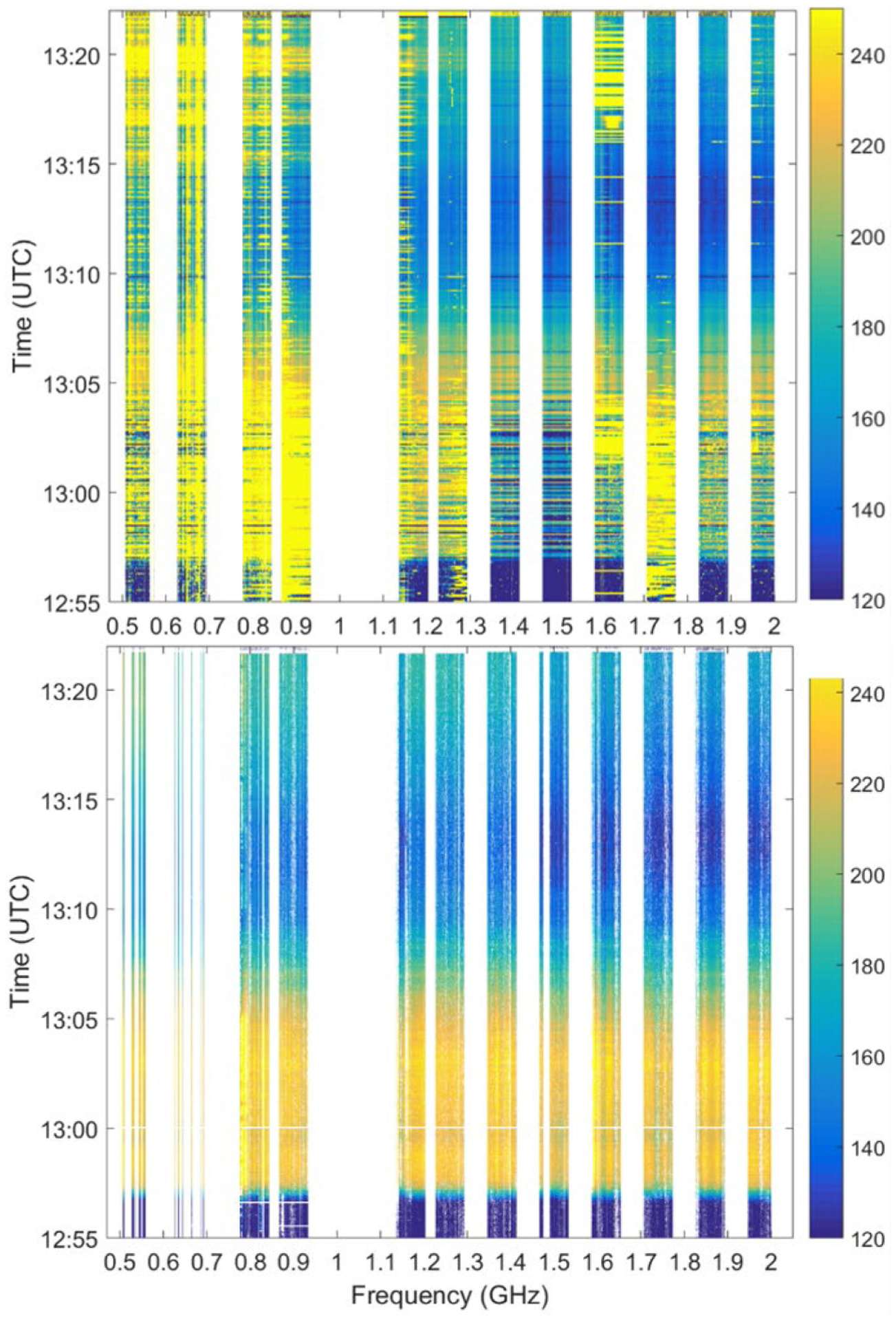
UWBRAD brightness temperature spectrogram (Kelvin) for a portion of the 2017 flight over the Greenland Ice Sheet near Thule Air Base before (upper) and after (lower) RFI processing.

UWBRAD was deployed in airborne observations of the northwestern Greenland Ice Sheet in 2016 and 2017, and across the Antarctic Ice Sheet in 2018. Fig. 8 illustrates sample calibrated brightness temperature spectrograms acquired by UWBRAD near Thule Air Base, Greenland, in 2017. Note that significant RFI was observed in this portion of the flight, presumably due to sources near the base and those operating aboard the aircraft. The lower plot shows that a combination of RFI processing strategies can allow the geophysical signatures of this portion of the ice sheet to be acquired at the cost of the loss of some portions of the observed bandwidth and integration time.

**Fig. 9.**
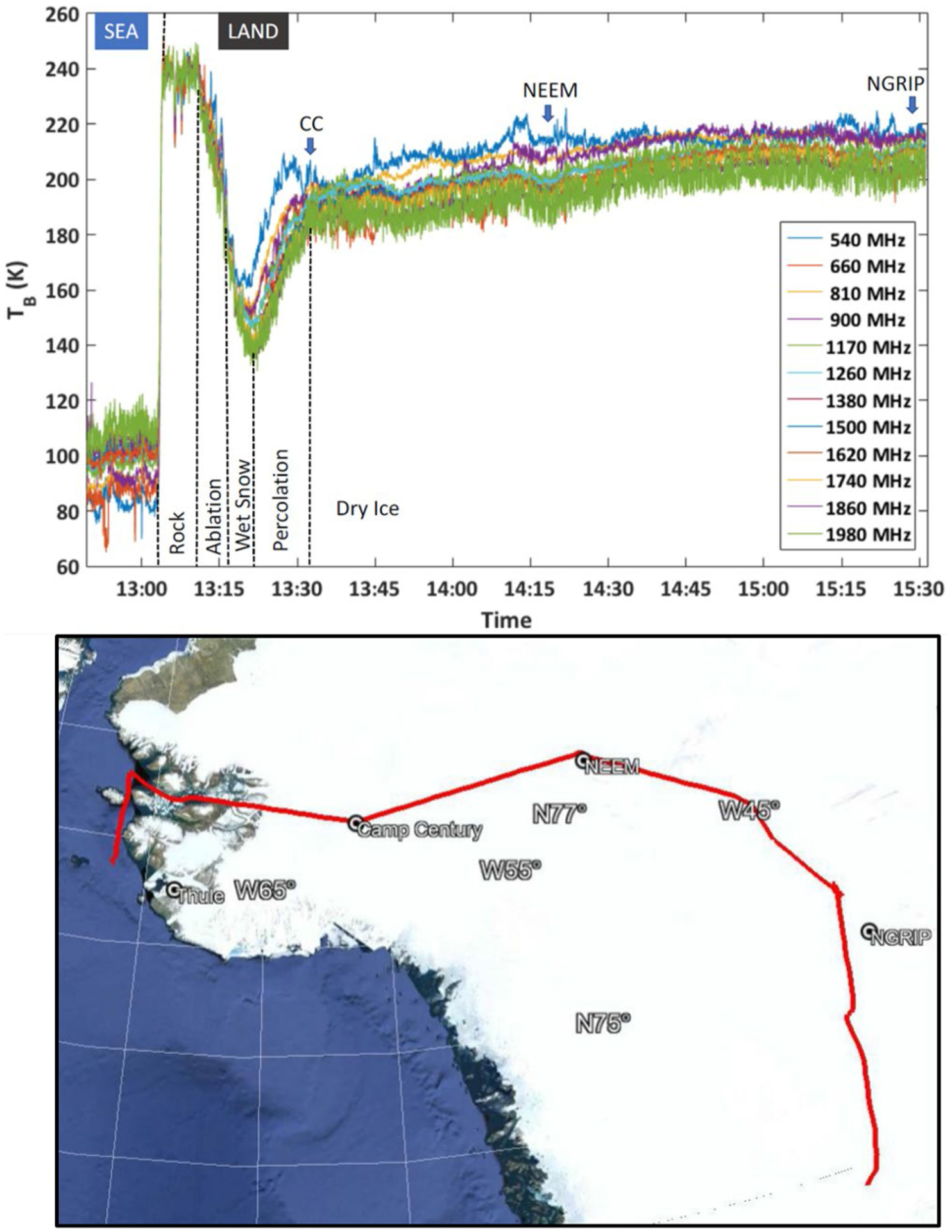
UWBRAD brightness temperatures in 12 channels (upper) for portion of 2017 flight over Greenland ice sheet (lower).

Fig. 9 plots UWBRAD nadir-looking right-hand circularly polarized brightness temperatures for a portion of the 2017 flight over the Greenland Ice Sheet (flight path in lower portion of figure) following an integration over the 512 subchannels of each frequency band (after RFI processing). The results show low brightness temperatures for a portion of the flight path over the sea surface, followed by higher values over the rocky shores of Greenland, and then a significant decrease within the percolation facies of the ice sheet where scattering loss is appreciable even at these frequencies [26], [28]. As the flight transitioned to the dry snow zone in the upper elevations of the ice sheet (between the Camp Century, NEEM, and NorthGRIP borehole sites), brightness temperatures showed smaller fluctuations.

**Fig. 10.**
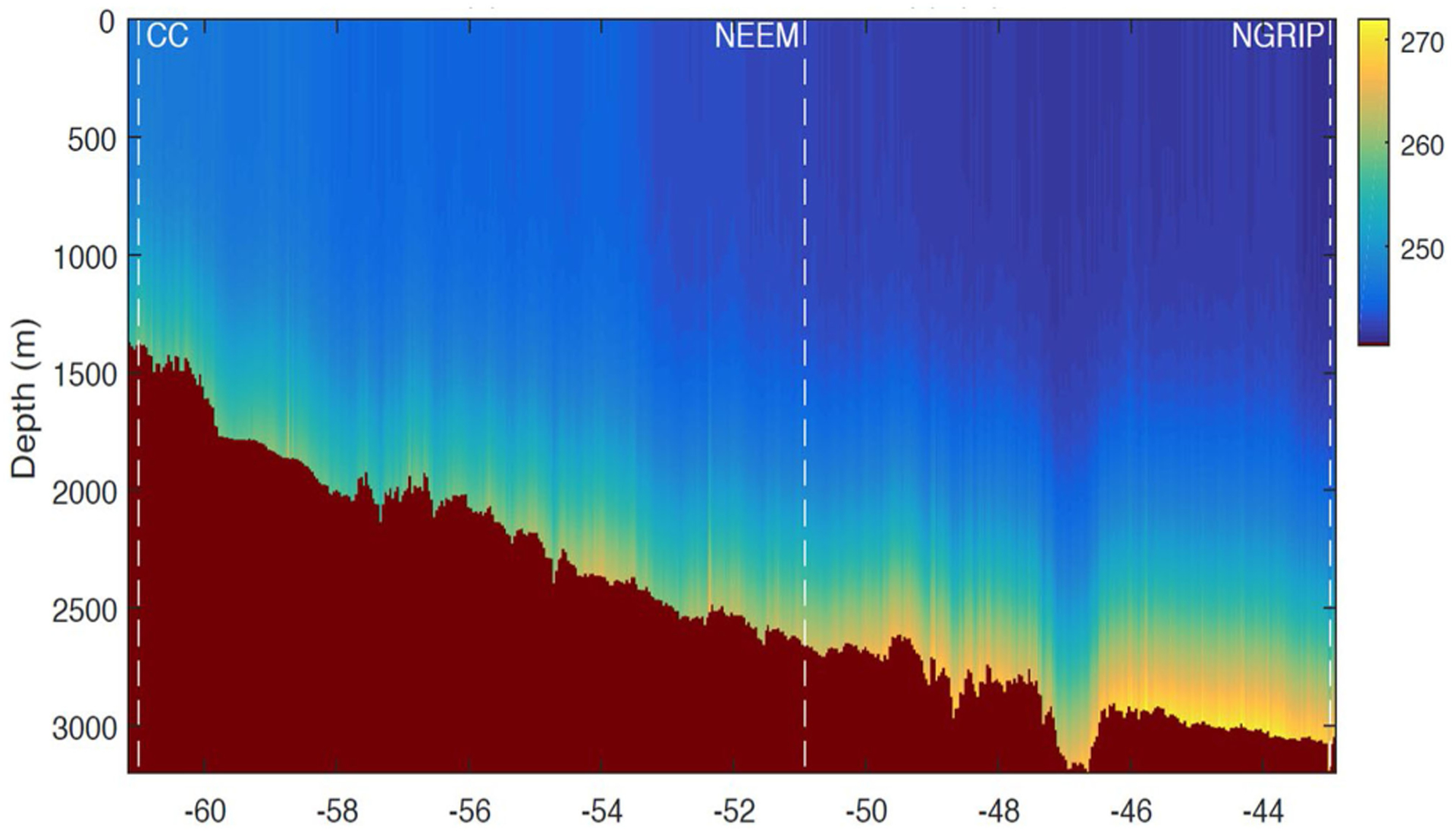
Temperature profiles (Kelvin) within Greenland ice sheet retrieved from 2017 UWBRAD observations versus longitude in degrees. Basal topography [102] shown in dark red.

Measurements between the borehole sites were then used to retrieve ice sheet temperature profiles (Fig. 10) by matching brightness temperatures predicted by a forward model and measured data [26]. The retrieval process also requires constraints on the density fluctuations within the ice sheet to be introduced; these constraints were obtained through use of the *in situ* temperature profile information available at the borehole sites. While this implies that the retrieved data are not independent of the *in situ* temperature information, the results between the borehole sites all show reasonable variations, as expected for ice sheet temperature profiles in this region.

Yardim *et al.* [26] further provide estimates of the error in the temperatures retrieved as a function of depth and position that show a significant reduction in uncertainty when compared to the *a-priori* estimates available.

These results demonstrate the utility of 500–2000 MHz brightness temperature observations for obtaining information on ice sheet internal temperatures and for discriminating between ice sheet facies. Ongoing studies are incorporating ancillary information on density properties from models or from other radar measurements. Results from the 2018 Antarctic deployment are also expected to be reported in future publications [103].

### B. Measurements of Sea Ice Properties

**Fig. 11.**
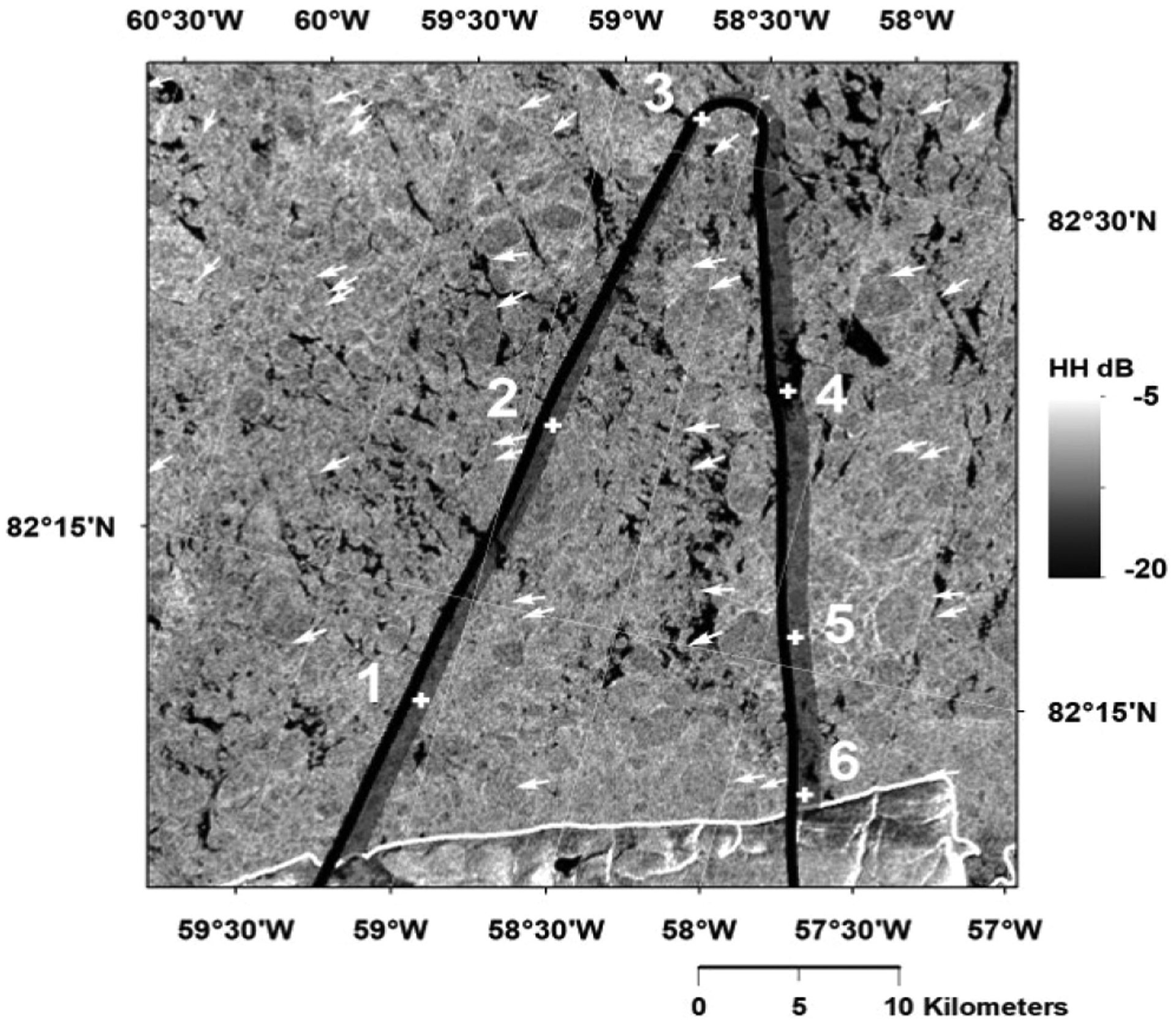
Flight path over Arctic sea ice in 2017 UWBRAD Greenland campaign including labels for six locations of interest, overlaid on ALOS-2 PALSAR SAR image.

**Fig. 12.**
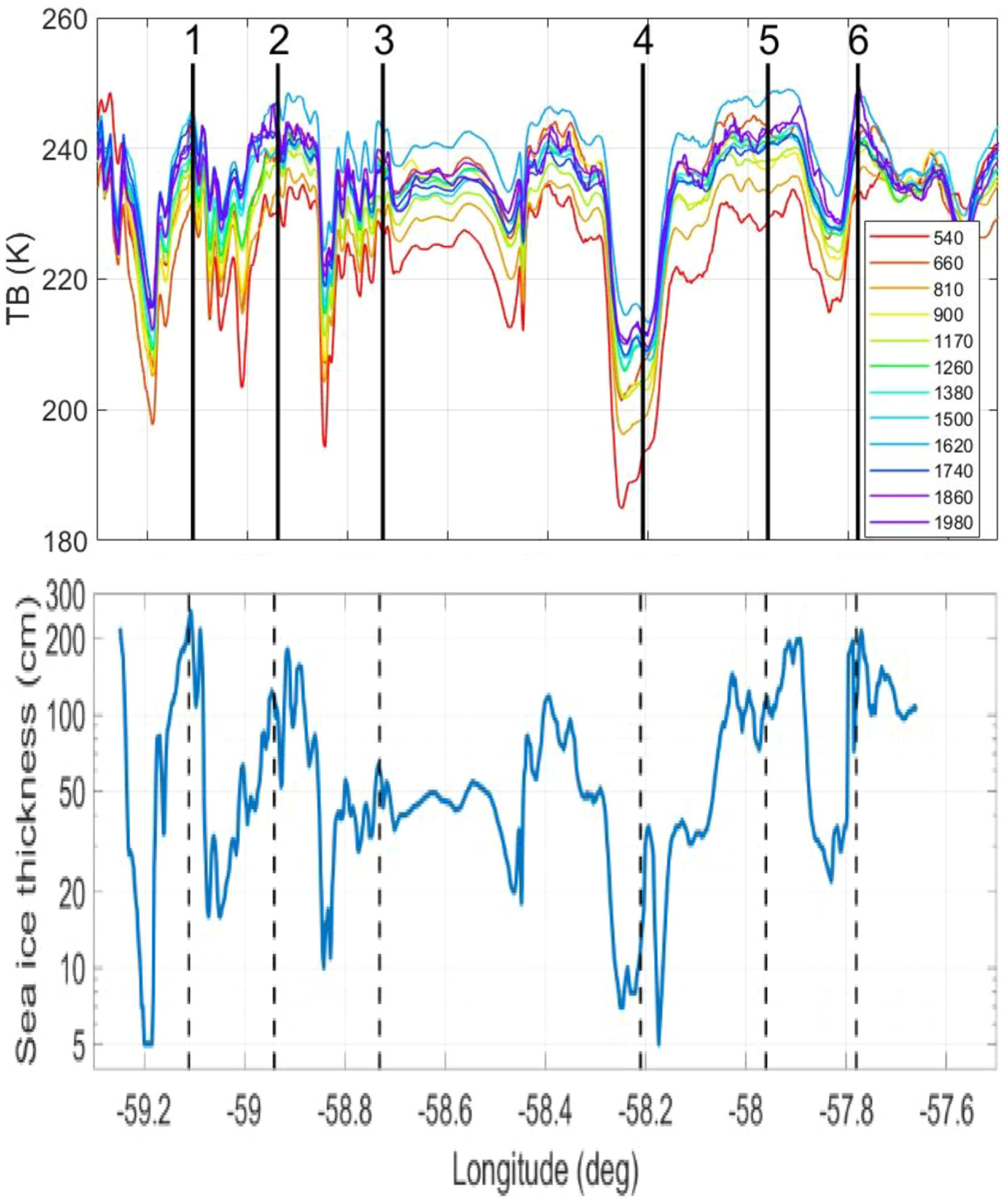
(Upper) UWBRAD 12 channel brightness temperatures over 2017 Greenland sea ice flight path shown in Fig. 11 (lower) sea ice thickness retrieved from UWBRAD observations; numbered locations as in Fig. 11.

The UWBRAD deployments to Greenland in 2017 and to Antarctica in 2018 also included observations over sea ice [22]. Fig. 11 illustrates the 2017 Greenland sea ice flight path; the corresponding brightness temperature observations are shown in the upper plot of Fig. 12. Six locations of interest are labeled in both figures. The flight path included a mixture of both “thin” and “thicker” ice due to dynamic activity in this region at the time of the experiment. The variation in brightness temperatures from “warmer” (i.e., thicker ice) to “colder” (thinner ice) is apparent, along with changes in the spectral signatures in each case. Unfortunately, no *in situ* information on ice thickness was available for this experiment at commensurate spatial scales, so that any retrievals obtained from the UWBRAD measurements can be assessed in terms of their plausibility only. Datasets from coastal sea ice observations in the 2018 Antarctic campaign have more extensive *in situ* ground truth and are currently under investigation.

Retrievals of sea ice thickness and salinity from these measurements were performed again through matching between measurements and a forward model for sea ice brightness temperatures. The latter was developed using standard models for incoherent emissions from a layered medium, with ice, air, and SSTs obtained from weather models [20]–[22], [54]–[57]. The required model for the dielectric constant of sea ice as a function of ice temperature and salinity was adapted from [57]. No evidence of brightness temperature oscillations in frequency was obtained (as would occur if the sea ice interfaces were very smooth [72]–[78]). This is not a surprising result given the high levels of sea ice roughness at both the ice–water and ice–air interfaces that can occur in dynamically evolving sea ice regions.

The lower plot of Fig. 12 illustrates the retrieved sea ice thicknesses obtained, and shows variations over plausible ranges of <10 cm for the “thin ice” cases to ~2 m for the thicker cases. The retrieved salinities (not shown) are also plausible, but in some cases appear to be higher than expectations. The source of this overestimation of salinity is currently under investigation, and may be related to snow layers overlying the sea ice. Model investigations of these effects are continuing to improve future retrievals. It is noted that the 2 m thickness retrieved appears to exceed the ~100 cm “saturation” level in Fig. 5, but such saturation levels are dependent on sea ice salinity and can approach 2 m or more with lower salinity multiyear ice.

A four-channel version of the UWBRAD instrument was deployed for *in situ* observations of sea ice as part of the Multidisciplinary drifting Observatory for the Study of Arctic Climate campaign from Sep. 2019 to Aug. 2020 [104], [105]. The extensive *in situ* information available on sea ice properties acquired during this campaign is providing further opportunities for improving understanding of the effect of sea ice structure and composition on 500–2000 MHz brightness temperatures.

### C. Soil Moisture Remote Sensing Experiments

Two airborne field experiments have been conducted in Australia demonstrating the capability of passive microwave soil moisture remote sensing at “P-band” (742–752 MHz) in comparison to L-band [14], [15]. Both P- and L-band brightness temperature observations were made with a spatial resolution of 75 m at multiple incidence angles using the polarimetric P-band multibeam radiometer (PPMR) and the polarimetric L-band multibeam radiometer (PLMR). The PPMR operates at 742–752 MHz having four dual-polarized beams with look angles of ±15° and ±45°, respectively, and a beam width of 30° × 30°. The PLMR operates at 1401–1425 MHz with six dual-polarized beams having looking angles of ±7°, ±21.5°, and ±38.5°, respectively, and a beamwidth of 17° × 15°. The calibrations of PPMR and PLMR were confirmed before and after each flight using the sky and a microwave absorber box as cold and warm targets, respectively, and an accuracy of better than 1.5 K was achieved. Intensive ground sampling of the top 5 cm soil moisture, vegetation water content, and surface roughness was also undertaken coincident with airborne measurements.

**Fig. 13.**
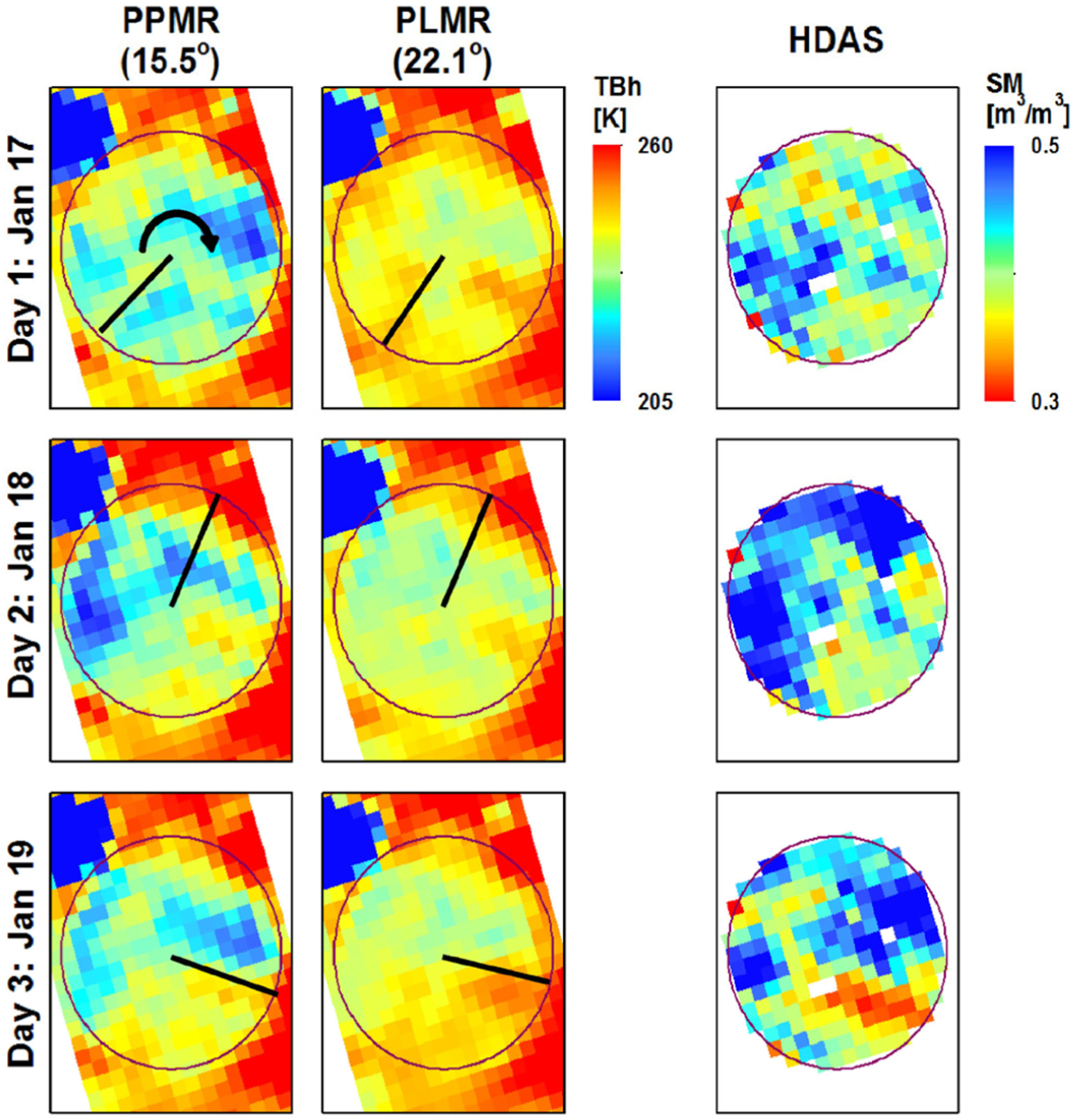
Maps of P- and L-band brightness temperature observations over the Cressy area at incidence angles of 15.5° and 22.1°, respectively, together with ground soil moisture measurements over the study area on three consecutive days. Black lines show the location of a center pivot irrigator at the time of sampling, with the arrow showing the direction of rotation (adapted from [14]).

Ye *et al.* [14] present the results of experiments conducted over a center pivot irrigated dairy farm, with radius of ~500 m at Cressy in Tasmania, Australia between the 17th and 19th of January 2017. The circular farm was dominated by pasture with different height and density, and with a reservoir located in the northwest. Fig. 13 shows brightness temperature observations at P- and L-bands (acquired within 1 h during morning flights) together with ground soil moisture measurements acquired later in the day gridded to 75-m resolution across the experiment period. Both P- and L-band brightness temperatures are found to decrease with higher soil moisture, but P-band brightness temperatures show a higher correlation to the soil moisture spatial pattern than those at L-band. This is expected to be due to a higher penetration depth at P-band and a greater sensitivity of P-band brightness temperature to soil moisture.

**Fig. 14.**
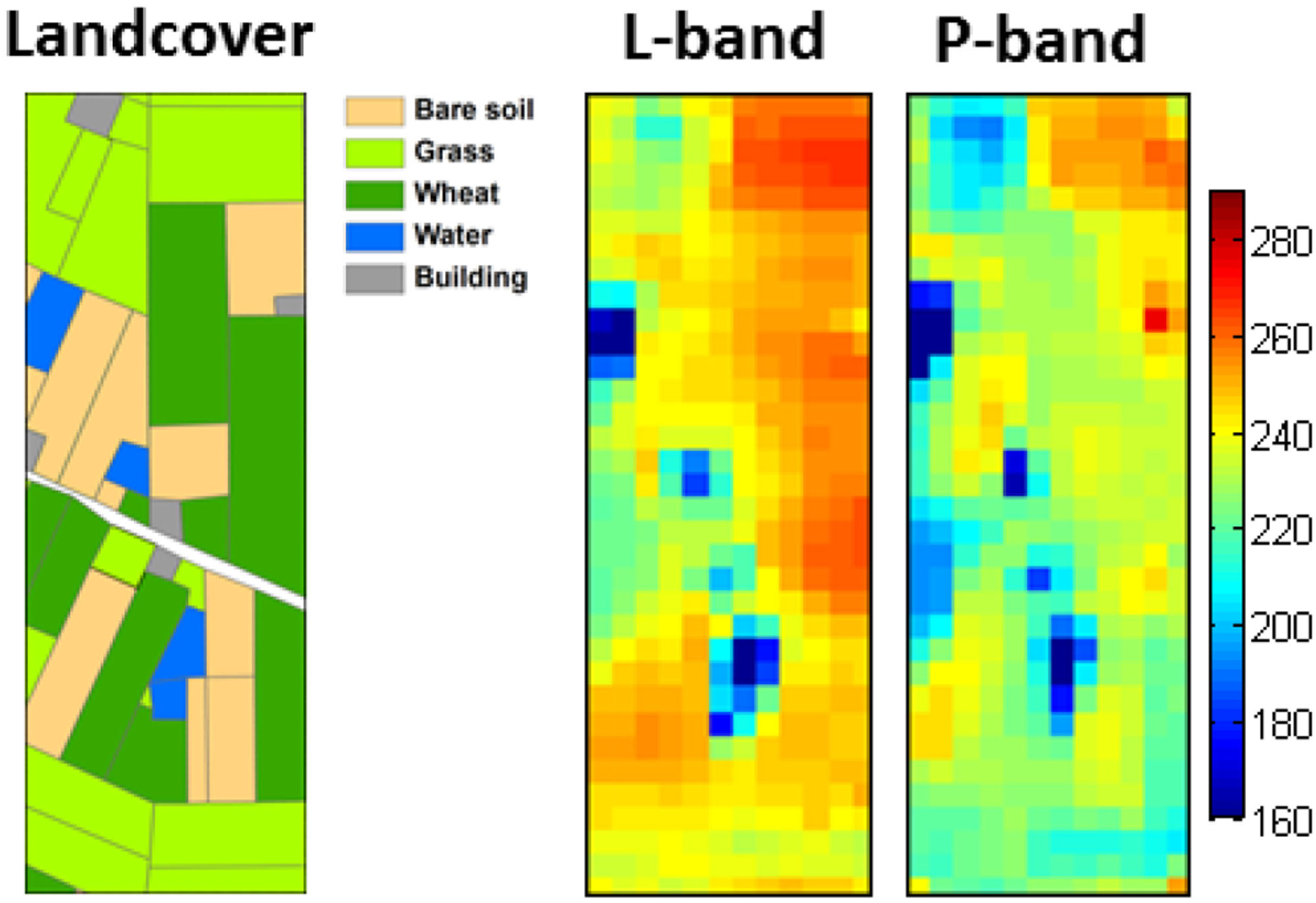
Land cover map with P-/L-band horizontally polarized brightness temperature images collected from 21° and 15°, respectively, over the Cora Lynn flight area on October 1st 2018 (adapted from [15]).

The P-band soil moisture remote-sensing capability was further studied over a heterogeneous cropping area of 900 m by 2550 m at Cora Lynn, located to the south east of Melbourne, Australia. A total of five flights were carried out every ~3 days during a two-week long airborne field experiment from October 1^st^–12th, 2018. Fig. 14 shows the land cover over the study area and brightness temperature maps at P- and L-band collected on October 1st. A similar spatial pattern was found between brightness temperature observations at both frequencies, with some subtle variations interpreted as due to the deeper sensing depth at P-band. In particular, P-band had lower brightness temperatures than L-band over agricultural fields, potentially due to higher penetration through vegetation.

**Fig. 15.**
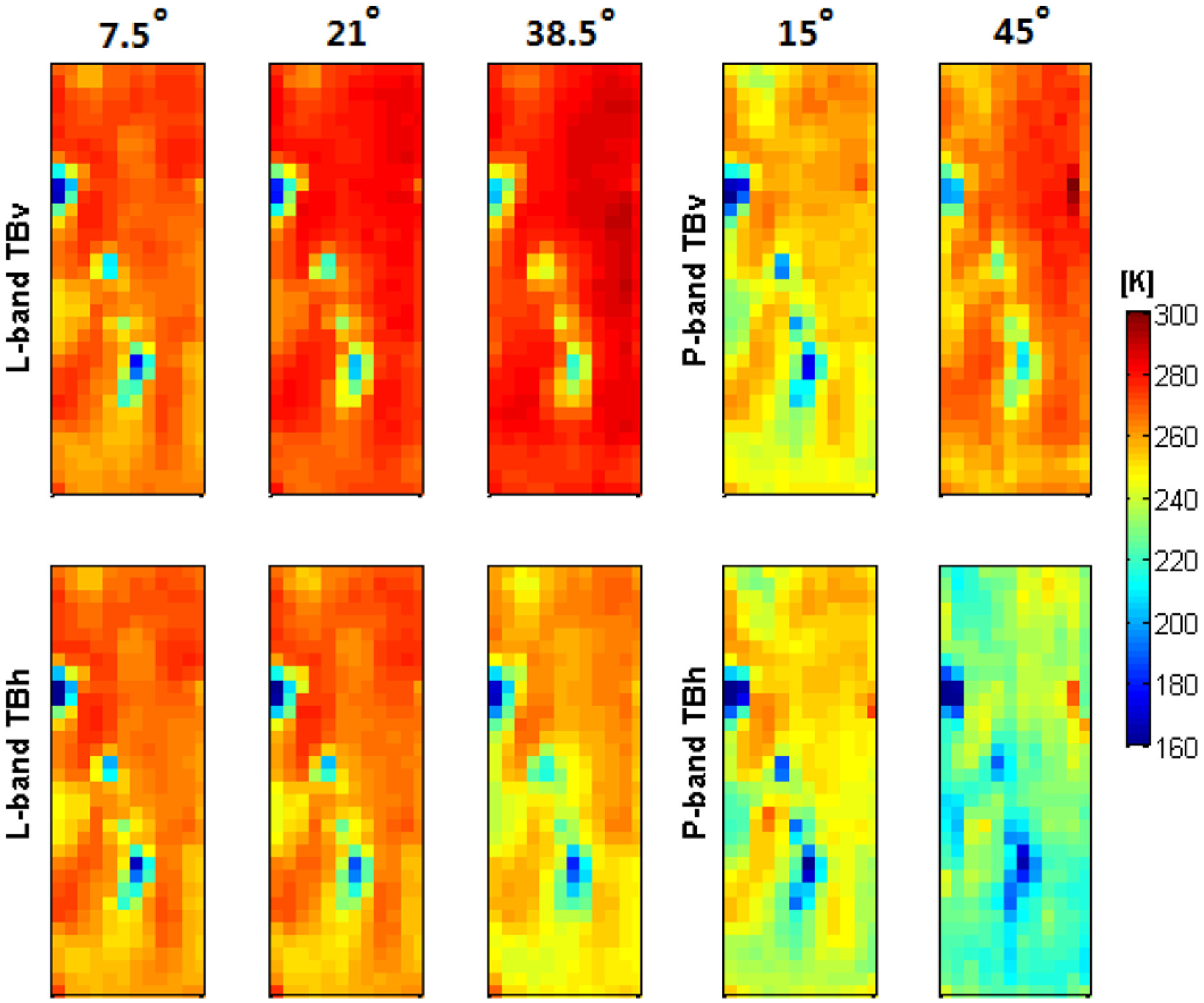
Dual-polarized brightness temperature images over the Cora Lynn flight area at multiple incidence angles (7.5°, 21°, and 38.5° for L-band; 15° and 45° for P-band) collected on October 12th 2018 (adapted from [15]).

Multiangle brightness temperature maps are shown in Fig. 15. The P-band brightness temperatures had a stronger angular response than those at L-band, especially in vertical polarization. It was found that the angular relationship at P- and L-bands varied under different land surface conditions. Consequently, the impact of vegetation water content and soil roughness on the multiangular P-band brightness temperature response is under further investigation.

### D. Mission Studies

Macelloni *et al.* [31] describe a proposal based on the approaches described here for the CryoRad mission formulated under the support of ASI (Italian Space Agency). The CryoRad proposal was submitted to ESA’s Earth Explorer 10 competition, and though not selected, received favorable reviews for its novelty and scientific maturity; the proposal has also been resubmitted to the Earth Explorer 11 competition. The CryoRad proposal’s goals were devoted to advancing cryospheric science by providing measurements of sea ice thickness and salinity, ice-sheet temperatures, SSS, and the status of permafrost. The mission concept was based on a low-frequency, wideband radiometer operating in the frequency range 400–2000 MHz. The proposed CryoRad antenna is a large deployable reflector whose diameter of ~12 m balances trade-offs between science performance and cost/complexity considerations. The proposed reflector antenna would include an antenna feed cluster having nine circularly polarized feed horns, and would be deployed in orbit. CryoRad’s polar orbit was designed for complete and continuous coverage of the poles, and would operate with a repeating ground track to revisit ground calibration sites and to provide regular repeat intervals. CryoRad would observe at nadir and with circular polarization to avoid Faraday rotation effects. The mission studies of [31] show that with a swath of 120 km and a field of view of 40 km at the lowest frequency, it would be possible to provide an average revisit time of three days at latitudes greater than 60° and ten days at the equator. The development of the CryoRad mission concept is continuing toward additional mission opportunities.

Dinnat *et al.* [16] report on science requirements and the technical definition of a next-generation spaceborne instrument for SSS and sea ice remote sensing. The new sensor is designed to improve salinity retrievals in cold waters to improve measurements in coastal regions, and to retrieve sea ice thickness. Science requirements were derived from a general ocean circulation model and observations reported in the literature with a special focus on coastal currents and river plumes. The study’s goals included the resolution of surface features as small as 20 km to enhance the capability to monitor SSS in coastal oceans and to detect SSS mesoscale variability in the world ocean. This requirement points to a 15-m class reflector antenna. Measurements in the cold waters of high latitudes drive the requirement for increased sensitivity to SSS, which can be achieved by using frequencies below 1000 MHz. Additional frequencies up to 7000 MHz were also considered to complement frequencies less than 2000 MHz to retrieve information about other environmental parameters such as surface roughness and SST.

**Fig. 16.**
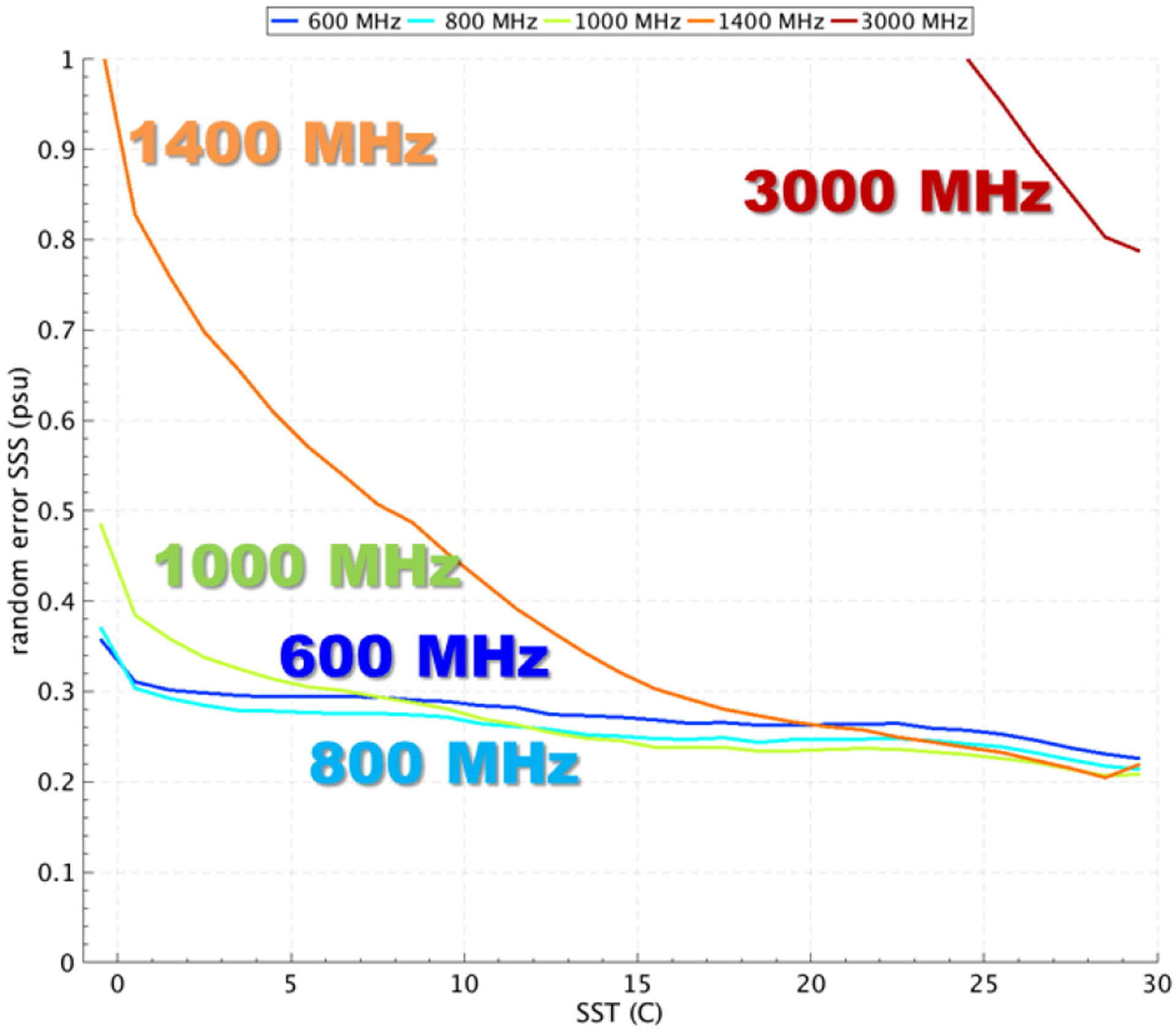
Simulated random error in satellite SSS retrieval as a function of SST using frequencies from 600 to 3000 MHz. Satellite and true SSS errors are averaged on daily maps at 0.5°×0.5° resolution in latitude and longitude [16].

**Fig. 17.**
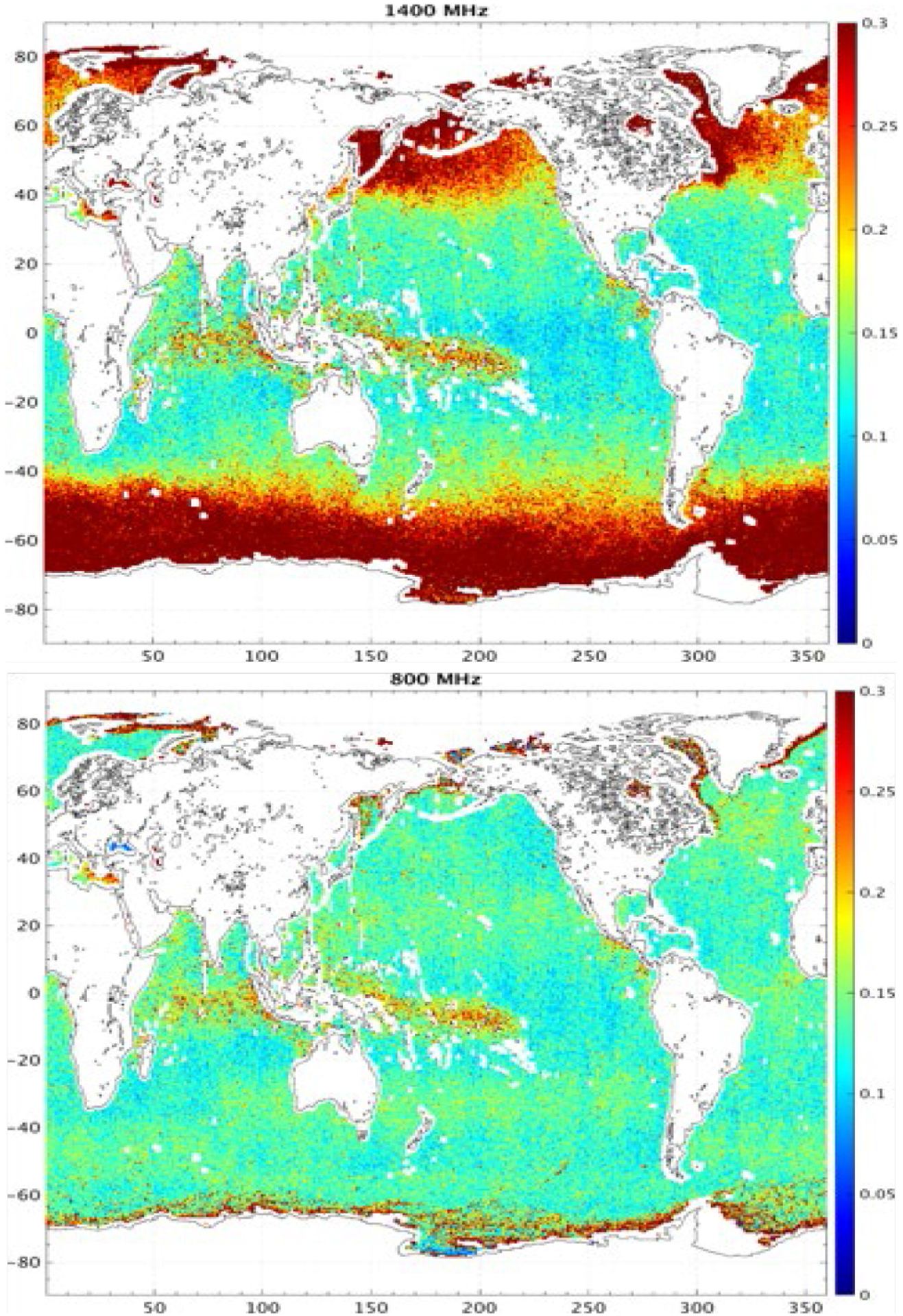
Maps of simulated random error in satellite SSS for retrievals using (top) 1400 MHz and (bottom) 800 MHz frequencies. Maps are derived from six month of weekly products at 0.5°×0.5° resolution in latitude and longitude [16].

The expected SSS retrieval performance was computed using a mission simulator based on a state-of-the art radiative transfer model (two-scale model with wind and dielectric parameterizations validated at low microwave frequencies) to predict ocean brightness temperatures over a period of six months at six frequencies (600, 800, 1000, 1400, 3000, and 5000 MHz). Spatiotemporal coverage was derived from the orbit parameters and sensor geometry of the SMAP L-band microwave radiometer. Random noise was added to the simulated brightness temperature measurements and on the *in situ* parameters (SST, wind speed and direction, SSS) used in the forward model. The model for the radiometer NEDT accounted for the expected hardware performance and included an increase in bandwidth at the higher frequencies. SSS retrievals were computed from the resulting noisy sensor data and noisy ancillary data and compared to the true SSS values that were input to the simulator. Simulated SSS errors (Fig. 16) show a substantial reduction at the lowest frequencies for waters 15°C and below, with a reduction by a factor 3 between 1400 and 600–800 MHz. A compromise between increasing the brightness temperature sensitivity to SSS and SST (a source of error) leads to an optimum frequency of ~800 MHz that slightly outperforms lower frequencies. A significant advantage of frequencies below 1000 MHz is the homogeneous performance across a range of SST values, improving on results using only 1400 MHz which suffer from regional and seasonal changes in performance as illustrated in the error maps of Fig. 17.

## V. Recommendations

The analyses and experiments reported in this article summarize the growing evidence of the potential for microwave radiometry from 500–1400 MHz to make a significant contribution to the future of Earth remote sensing. A growing international community of researchers is exploring these concepts, and it is reasonable to expect continued progress in the near future. Continued research is recommended particularly in the following areas:

1. Assessments of spectrum availability for spaceborne observations through both measurements and analysis, including surveys of licensed emitters internationally and estimations of the impact of these emitters on spaceborne microwave radiometers.
2. Ultra-wideband antenna systems, including feeds, suitable for space-based operation that can provide spatial resolutions of ~50 km or better from orbit while minimizing mass and volume requirements.
3. Ultra-wideband or multiple-frequency narrow-band receiver architectures and calibration procedures to provide high performance while minimizing size, weight, and power consumption.
4. Continued development and assessment of subsystems for detecting and filtering RFI in this frequency range.
5. Models to predict brightness temperature signatures from 500–1400 MHz for geophysical media, including refined models for the dielectric constant of sea water, sea ice, soil, permafrost, and meteoric ice in this frequency range. A recent experiment to improve knowledge of the dielectric properties of ice sheets in the 500–1400 MHz region by analyzing a 100-m ice core extracted at Dome-C Antarctica is noted as an example [106]. Continued improvement in characterizing the influence of the spatial variation of ice-sheet density (as in [26], [69]–[71]) is also recommended.
6. Continued development of geophysical parameter retrieval algorithms to improve performance and to specify any required ancillary datasets or remote sensing observations (for example, SAR observations of sea ice to address inhomogeneous ice types within a radiometer footprint).
7. Continued ground-based and airborne demonstrations of geophysical remote sensing over a wider range of target types and conditions with supporting *in*-*situ* measurements.
8. Studies of the science and application impacts of new information on ice sheet internal temperatures, sea ice thickness, cold water SSS, soil moisture, permafrost, and other geophysical products that may be enhanced by these measurements, including constraining and improving models and predictions.

## VI. Conclusion

The potential of microwave radiometry from 500–1400 MHz for improving the remote sensing of land, sea, and ice surfaces clearly motivates the continuing development of this technology. The increased penetration through geophysical media available at these frequencies, the increased sensitivity to SSS, the decreased sensitivity to scattering inhomogeneities and surface roughness, and the potential to sense deeper subsurface temperatures all provide distinct advantages for lower frequency microwave radiometry as compared to existing sensors. Investigations are continuing internationally with the goal of resolving the remaining questions, expanding the datasets and demonstrations available, and advancing toward operation in space.

## Figures and Tables

**Table I:** Properties of the UWBRAD Microwave Radiometer

Frequency	0.5–2 GHz, 12×~100 MHz channels
Spatial Resolution	600 m×600 m (500 m platform altitude)
Polarization	Right-hand circular
Observation Angle	Nadir
Antenna Gain/HPBW	10 dB / 60°
External Calibration	Ocean measurements
Internal Calibration	Reference load and noise diode
Noise Equivalent d*T*	~1K in 100 ms (each channel)
Interference Management	Software-defined RFI detection/mitigation
